# Effects of Screen-Based Leisure Time on 24 Subsequent Health and Wellbeing Outcomes: A Longitudinal Outcome-Wide Analysis

**DOI:** 10.1007/s12529-024-10307-0

**Published:** 2024-07-18

**Authors:** Pedro A. de la Rosa, Richard G. Cowden, Joseph A. Bulbulia, Chris G. Sibley, Tyler J. VanderWeele

**Affiliations:** 1 Institute for Culture and Society, University of Navarra, Campus Universidad de Navarra, sin número, 31009 Pamplona, Spain; 2 Department of Epidemiology, Harvard T.H. Chan School of Public Health, Boston, MA USA; 3 Human Flourishing Program, Institute for Quantitative Social Science, Harvard University, Cambridge, USA; 4 School of Psychology, Victoria University of Wellington, Wellington, New Zealand; 5 School of Psychology, University of Auckland, Auckland, New Zealand

**Keywords:** Internet, Epidemiology, Video games, Social networks, Health, Wellbeing

## Abstract

**Background:**

Previous research has shown that screen-based leisure time is related to physical and mental health, relationships, and prosocial behaviors. However, it remains unclear whether screen-based leisure time causally affects wellbeing, as previous studies have relied on cross-sectional data, focused on one type of media use (e.g., social media, video games, or internet), or assessed a narrow set of outcomes.

**Method:**

We used three waves (2016, 2017, 2019) of national longitudinal data from the New Zealand Attitudes and Values Study to investigate the effects of screen-based leisure time on 24 parameters of wellbeing (*n* = 11,085). We operationalized screen-based leisure as the sum of time spent browsing the internet, using social media, watching/reading the news, watching videos, and playing video games. We followed the outcome-wide analytic design for observational data by performing a series of multivariable regression models estimating the effect of screen-based leisure time on 24 wellbeing outcomes and assessed potential unmeasured confounding using sensitivity analyses.

**Results:**

In our primary analysis with the total sample, total screen-based leisure time was associated with a very modest decrease in body satisfaction and a very modest increase in body mass index. Possible evidence of associations was found with increases in number of hours spent exercising and volunteering each week, as well as decreases in number of average daily hours of sleep, self-control, and subjective health.

**Conclusion:**

Screen-based leisure time has the potential to affect health and wellbeing. Results are discussed in light of the high prevalence of screen-based leisure time.

**Supplementary Information:**

The online version contains supplementary material available at 10.1007/s12529-024-10307-0.

## Introduction

During the last two decades, digital technologies have permeated most at-home activities. Although watching television or playing video games could be considered leisure activities that are distinct from internet use, nowadays it is common for video games or streaming on-demand services to require a stable internet connection. Social media platforms (e.g., Twitter, YouTube) have evolved to be places where people share content with friends and find news on the internet, coexisting with classical media such as newspapers, cable television, and radio [1]. Therefore, the scope of research on the effects of screen-based activities has expanded with the exponential growth of digital technologies.

Existing literature on the relationship between screen-based activities and health and wellbeing covers different topics. First, research has explored the effect of the sedentary nature of screen-based activities. These activities are usually done while sitting for many hours combined with snacking, increasing body mass index or other cardiovascular risk factors [2–4]. There is evidence that time spent watching television is related to higher body mass index, even after controlling for physical exercise and food intake [5]. Second, the effect of screen-based activities may change according to the cognitive or physical engagement of a person when using a device. Active screen-based activities, such as playing video games, working with computers, or chatting on social media, require concentration. In contrast, other passive screen-based activities, such as watching videos, typically do not. Negative mood changes are associated with passive screen activities, but not with active ones [6]. However, the literature is mixed in this regard, with both positive and negative effects for both active and passive internet use [7]. Third, exposure to media messages can shape users’ attitudes [8]. Research has shown that some sexual content or drug depictions in video games or movies can shape normative beliefs among youth, making it easier to adopt attitudes favorable to those behaviors [9, 10]. More broadly, social media has the potential to influence political polarization [11]. Fourth, excessive or problematic internet use or related activities may impact health and wellbeing [12–14]. Excessive and uncontrolled use of the internet, social media, or video games (and other related activities) can take time away from healthier leisure or social activities and are associated with depression and anxiety symptoms [15].

**Fig. 1:**
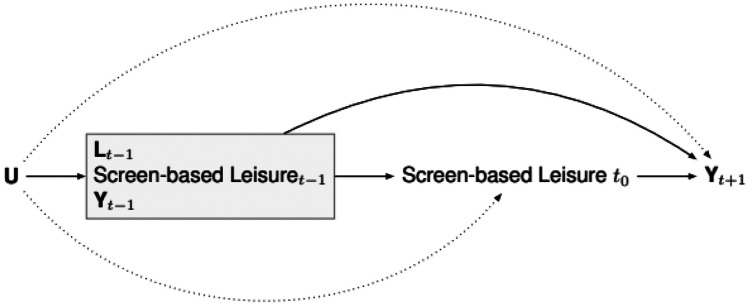
Directed acyclic graph summarizing outcome-wide analytic framework

Although prior research has provided helpful insight into the potential implications of screen-based leisure time for health and wellbeing, several gaps remain. Most of the existing research in this area has relied on cross-sectional data, focused on one type of media use (e.g., social media, video games), and assessed one or few outcomes with small-sized samples [16, 17]. These drawbacks hinder, on the one hand, the ability to establish causal conclusions about the association between screen-based leisure time and health and wellbeing. On the other hand, it is difficult to compare such studies because they often use different variables and biases can vary from study to study. To fill some of these gaps in knowledge and improve the robustness of current evidence on this topic, cohort studies are needed that consider various types of screen-based activities and their relationship with different parameters of health and wellbeing. The New Zealand Attitudes and Values Study (NZAVS) is a national diverse longitudinal probability study of New Zealanders (http://www.nzvalues.org/) that is ideally suited for investigating the causal effects of screen-based activities on health and wellbeing. First, the NZAVS contains a variety of variables that capture internet use, lifestyles, personality, wellbeing, and personal values (among others). Second, the study contains information about different forms of screen-based activities, including browsing the internet, watching videos, watching news, use of social media, and playing computer games. Third, 94% of New Zealand’s population had internet access before 2020 [18], making this database appropriate for studying the effects of leisure time spent engaged in different screen-based activities. Fourth, the study follows the same individuals over time, which allow us to identify the effects of increases in the levels of screen time on a set of outcomes. Fifth, NZAVS contains rich indicators of potential confounding variables: Through rigorous methods for minimizing confounding, the NZAVS allows causal identification for the specific effects of screen-based leisure time across a spectrum of health and wellbeing outcomes under the assumption that there is no unmeasured confounding or, if any, its implications are quite negligible. Figure 1 presents a causal directed acyclic graph that clarifies the outcome-wide approach for causal inference in a longitudinal study for which there is potential selection bias from non-response.

The exposure is hours of screen-based leisure time. Y denotes an outcome. L denotes the rest of covariates. By multiplying imputing missing responses (on missing-at-random assumptions), we adjust for selection biases arising from non-response. Notice that by adjusting for prior exposure at t − 1, we reduce the threat of unmeasured confounding, U. To explain away an observed exposure-outcome association, the unmeasured confounder would have to be associated with both the outcome and the baseline exposure, independent of the prior level of exposure [19]. Such adjustment enables “incidence” exposure rather than “prevalence” exposure, limiting confounding biases from the effects of previous exposures on the outcome of interest. Moreover, by adjusting for past confounders at t − 1, we control for common causes of the exposure at t − 0 and the outcome at t + 1. Among the strongest of such confounders may be past health or wellbeing outcomes, the inclusion of which at baseline (Y_t−1_) provides further additional confounding control. Finally, because we cannot ensure that all unmeasured confounders have been controlled (the dotted lines in the graph). For example, if an unmeasured confounder affects the measured confounders at t − 1, there will be bias. Suppose the unmeasured confounder affects non-response. In that case, the missing-at-random assumption is incorrect, and the statistical association will provide a biased estimate of the actual causal association.

For this reason, we provide sensitivity analyses using *E*-values to assess the minimum level of association needed for an unmeasured confounder to have with both the exposure and the outcome to explain away that observed association [20]. Note: to retain focus on our identification strategy, we omit arrows from past exposures to future exposures and past outcomes to future outcomes.

Drawing on the strengths of the NZAVS, there were two main objectives of the present study. First, we estimated the effect of total time invested in screen-based activities (browsing the internet, watching videos, watching or reading the news, using social media, and playing video games) on a wide set of health and wellbeing outcomes (e.g., psychological wellbeing, health, social relationships, health and prosocial behavior) among adults. Second, we assessed the impact of different screen-based activities on health and wellbeing outcomes according to generational categories. We split the sample into subsamples of younger participants (younger than 40 years) and older participants (40 years or more). This generational cut-off point was chosen because it is commonly applied in the empirical literature [21] and because the two groups differed in lifetime exposure to the internet. The younger subsample consists mostly of millennials and centennials, whose internet exposure started during childhood or adolescence. In contrast, older individuals (generation X or older) had to learn and adopt digital technologies during adulthood. We expected the health and wellbeing consequences of screen-based leisure time to be greater in the younger subsample, because this group tends to have higher engagement with internet-related technologies involving screens.

## Method

In the present research, we followed the outcome-wide framework for analyzing longitudinal data. Contrary to traditional studies that often focus on multiple exposures with one or a few main outcomes, outcome-wide studies analyze the relationship of one exposure of interest with numerous outcomes [19, 22]. This allows researchers to compare the effect of a single exposure on a wide range of outcomes within the same study, which can facilitate the design of health policies by considering the effect of the exposure on different aspects of health and wellbeing.

### Sample

The NZAVS is a 20-year longitudinal national probability study of social attitudes, personality, and health outcomes with a representative sample of more than 1% of all New Zealanders. The study aims to provide information about (1) how the personality, attitudes, and values of New Zealanders may be changing over time; and (2) how changes in attitudes and values may relate to changes in health and wellbeing over time. In this study, we used data from waves 8 (2016), 9 (2017), and 11 (2019).

Participants were invited to complete either an online or a paper-based survey if they did not provide an email address. A prize draw was offered for participants in all waves to encourage cohort retention, and non-respondents were followed up by email and phone on multiple occasions. Full details about the recruitment and retention of the participants can be found on the NZAVS website (http://www.nzvalues.org). The initial sample of wave 8 included 21,936 participants from previous waves (*n* = 13,781) and new participants from a booster sample taken from the New Zealand Electoral Roll (*n* = 7667). After 3 years of follow-up, 11,085 participants from the wave 8 sample had responded to both waves 9 and 11. In wave 11, a total of 2586 participants (23.33% of the sample) filled out the survey after 25 March 2020, when the COVID-19 pandemic government lockdown measures started in New Zealand. Among these three waves, 3013 (27.2%) participants completed a paper-based survey in each wave, 2870 (25.9%) completed an online survey in each wave, and 5202 (46.9%) completed either paper-based or online survey across the waves.

### Measures

Outcomes, exposures, and covariates for the present study are summarized below. A detailed list of the study variables can be found in Table S1.

#### Exposures

The main exposure was the total amount of screen-based leisure time. Participants from the NZAVS cohort were asked about the weekly hours spent in the following activities: browsing the internet, watching television/Netflix/movies, using social media (e.g., Facebook), watching/reading the news, and playing computer/video games. Total screen-based leisure time was computed as the sum of the time of these five activities. Besides the total screen-based leisure time variable, we additionally used time spent in each of the five screen activities as individual exposures in further analysis.

#### Outcomes

A range of outcome variables addressing the following domains was assessed: psychological wellbeing (life satisfaction, self-esteem), social wellbeing (sense of community, sense of belonging, social support), social factors (having been cyberbullied, engaged or entered a civil union, divorced or separated, or been assaulted, harassed or attacked), character strengths and prosocial behavior (amount of charity donations, weekly hours of volunteering, self-control), physical health (subjective health, average daily hours of sleep, body mass index, body satisfaction, recent diagnoses of circulatory diseases, recent diagnoses of musculoskeletal diseases, and consequences of external causes), mental health (psychological distress, diagnoses of mental health disorders), and health behaviors (alcohol consumption, current smoking, and weekly hours of exercise). Means and standard deviations for all continuous outcomes are presented in Table S2.

#### Covariates

We adjusted for confounding following the modified disjunctive cause criterion [23]: all models adjusted for pre-baseline (wave 8, T_0_) covariates that may be related to screen-based leisure time in the exposure wave (wave 9, T_1_) or to health and wellbeing in the outcome wave (wave 11, T_2_), or to both the exposure and one or more outcomes, either directly or by proxy, and excluded any known instrumental variable. This set of covariates included (1) demographic indicators (age, gender, ethnicity, born in New Zealand, resident in urban area, sexual orientation, religion, academic degree, New Zealand socioeconomic index, occupation, income attribution, relationship status, number of children, education level), (2) weekly time in other activities (housework, commuting, putting on cosmetics, working), (3) personality (neuroticism, openness, conscientiousness, extraversion, agreeableness), and (4) beliefs (political orientation, locus of health control).

To identify the causal effect of total screen-based leisure time in the exposure year (T_1_) on dimensions of health and wellbeing (T_2_), our statistical models adjusted for past health and wellbeing outcomes, past screen-based leisure time, and a rich array of pre-baseline confounders (T_0_). We used *E*-values to assess the sensitivity of results to unmeasured confounding. To adjust for selection bias, we used the chained equations multiple imputation method to impute missing data on all study variables with five imputed datasets [24].

### Statistical Analysis

Statistical analyses were performed using STATA, version 17 [25]; tests of statistical significance were 2-sided.

#### Primary Analysis

For the primary analysis, we performed a series of multivariable regression models to assess the effect of total screen-based leisure time at T_1_ on the 24 outcomes at T_2_ (one outcome at a time). We repeated these models for time spent in each type of the five screen activities at T_1_ on the 24 study outcomes at T_2_ (one activity and one outcome at a time). Thus, we performed six different outcome-wide analyses by running 24 models for each of the six exposures at T_1_. All models controlled for all covariates, the prior value of the exposure, and prior values of all outcomes assessed at T_0_.

Logistic regression models were used for binary variables with a prevalence < 10%, generalized linear models (with a log link and Poisson distribution) were used for binary outcomes with a prevalence ≥ 10%, and linear regression models were used for continuous variables. All continuous variables were standardized (that is, the unit of each variable is expressed in standard deviations) to allow for effect sizes to be compared across outcomes.

Since there is no standardized approach to correct for multiple testing, we report *p*-value cut-offs both before and after applying a Bonferroni correction (*p*-value threshold after Bonferroni correction = 0.05/24 = 0.002). Continuous *p*-values for all analyses can be found in the Online Supplement.

#### Secondary Analyses

To assess the effect of total screen-based leisure time and time spent in the different screen-based activities on the outcomes according to age groups, we split the sample into two generational categories: individuals less than 40 years of age and those aged 40 years or older. Then, all the multivariable models from the primary analyses were repeated for both subsamples.

#### Sensitivity Analyses

We performed four sensitivity analyses. First, we categorized total screen-based leisure time into quartiles. We replicated all the primary and secondary analyses using this categorical variable as the main exposure. In this way, we studied the effects of these activities without assuming a linear relationship between the variables. Second, we evaluated the robustness of the results in the primary and secondary analyses by calculating *E*-values. An *E*-value indicates the minimum level of association needed for an unmeasured confounder to have with both the exposure and the outcome to explain away that observed association [20]. Third, we replicated the primary and secondary analyses by additionally controlling for survey response format among the participants across the three waves (paper-based surveys only, online surveys only, or a mixture of both). Fourth, we replicated the primary and secondary analyses by excluding participants who completed the wave 11 survey after the date the severe lockdown was implemented in New Zealand due to the COVID-19 pandemic (25 March 2020). In order to limit or control transmission of SARS-CoV-2, the New Zealand government implemented several containment measures (e.g., travel restrictions, social distancing requirements). Research suggests that lockdowns affected people’s daily lives and wellbeing [26, 27], and therefore it may be important to explore whether the pattern of associations in the primary analysis might have been affected by the timing of participation during wave 11.

## Results

The distribution of participants’ pre-baseline characteristics within the overall sample and by quartiles of total screen-based leisure time is shown in Table 1. Participants had a mean pre-baseline age of 51.9 years (SD = 13.6); most of them were female (63.0%), from New Zealand (20.2%), of European ethnicity (84.4%), and resided in urban areas (64.1%). The mean amount of time spent on all five screen-based leisure activities was 33.1 h/week (SD = 24.3). The activities that participants reported spending the most time engaging in were browsing the internet (mean = 12.5 h/week, SD = 14.1) and watching videos (mean = 10.7 h/week, SD = 9.0); the activity with the lowest mean time of engagement was playing video games (mean = 1.3 h/week, SD = 4.2).

**Table 1:** Pre-baseline characteristics and prior outcome values in 2016 by quartiles of screen use in 2017

		**Groups of screen-based leisure time**				
	**Total sample**	**Quartile 1** (< 18 h)	**Quartile 2** (18 to 28 h)	**Quartile 3** (29 to 42 h)	**Quartile 4** (> 43 h)	***p*-value**
	***n* = 11,085**	***n* = 2802**	***n* = 2915**	***n* = 2597**	***n* = 2915**	
Age, *M* (*SD*)	51.9 (13.6)	52.3 (12.2)	52.0 (13.0)	52.8 (13.7	50.4 (15.1)	< 0.001
Gender, *n* (%)						< 0.001
Female	6981 (63.0)	1855 (66.2)	1845 (63.3)	1581 (60.9)	1700 (61.3)	
Male	4069 (36.7)	942 (33.6)	1065 (36.5)	1008 (38.8)	1054 (38.0)	
Not applicable	35 (0.3)	5 (0.2)	5 (0.2)	8 (0.3)	17 (0.6)	
Ethnicity, *n* (%)						< 0.001
New Zealand European/Pakeha	9353 (84.4)	2355 (84.0)	2501 (85.8)	2237 (86.1)	2260 (81.6)	
Asian	323 (2.9)	84 (3.0)	73 (2.5)	70 (2.7)	96 (3.5)	
Maori	996 (9.0)	246 (8.8)	240 (8.2)	206 (7.9)	304 (11.0)	
Pacific	172 (1.6)	41 (1.5)	41 (1.4)	37 (1.4)	53 (1.9)	
Born in New Zealand, *n* (%)						< 0.001
No	2238 (20.2)	535 (19.1)	575 (19.7)	516 (19.9)	612 (22.1)	
Yes	8846 (79.8)	2266 (80.9)	2340 (80.3)	2081 (80.1)	2159 (77.9)	
Area, *n* (%)						< 0.001
Urban	7111 (64.1)	1689 (60.3)	1891 (64.9)	1669 (64.3)	1862 (67.2)	
Rural	3875 (35.0)	1092 (39.0)	1001 (34.3)	911 (35.1)	871 (31.4)	
Not applicable	99 (0.9)	21 (0.7)	23 (0.8)	17 (0.7)	38 (1.4)	
Sexual orientation, *n* (%)						< 0.001
Heterosexual	9636 (86.9)	2416 (86.2)	2581 (88.5)	2276 (87.6)	2363 (85.3)	
Not heterosexual	715 (6.5)	138 (4.9)	155 (5.3)	173 (6.7)	249 (9.0)	
Not applicable	734 (6.6)	248 (8.9)	179 (6.1)	148 (5.7)	159 (5.7)	
Religion, *n* (%)						< 0.001
No religion	6792 (61.3)	1612 (57.5)	1776 (60.9)	1633 (62.9)	1771 (63.9)	
Christian	3653 (33.0)	997 (35.6)	990 (34.0)	825 (31.8)	841 (30.4)	
Other religion	640 (5.8)	193 (6.9)	149 (5.1)	139 (5.4)	159 (5.7)	
Religion identification, *M* (*SD*)	4.7 (2.0)	5.0 (2.0)	4.7 (2.1)	4.6 (2.0)	4.5 (2.0)	< 0.001
Academic degree, *n* (%)						< 0.001
Lower than intermediate high education	4022 (36.3)	1029 (36.7)	975 (33.4)	944 (36.3)	1074 (38.8)	
Intermediate high education diploma	1520 (13.7)	390 (13.9)	422 (14.5)	374 (14.4)	334 (12.1)	
Bachelor Degree	2886 (26.0)	726 (25.9)	762 (26.1)	661 (25.5)	737 (26.6)	
Postgraduate or higher	2632 (23.7)	642 (22.9)	752 (25.8)	614 (23.6)	624 (22.5)	
Occupation, *n* (%)						< 0.001
Clerical/administration	1241 (11.2)	297 (10.6)	308 (10.6)	296 (11.4)	340 (12.3)	
Manager	1674 (15.1)	436 (15.6)	448 (15.4)	406 (15.6)	384 (13.9)	
Proffesional	3921 (35.4)	1017 (36.3)	1078 (37.0)	894 (34.4)	932 (33.6)	
Other^d^	2243 (20.2)	633 (22.6)	589 (20.2)	504 (19.4)	517 (18.7)	
Unreported	2003 (18.1)	419 (15.0)	492 (16.9)	496 (19.1)	596 (21.5)	
NZ socioeconomic index, *M* (*SD*)	55.4 (15.9)	55.2 (16.7)	56.1 (16.0)	55.3 (15.6)	55.1 (15.4)	0.083
Income attribution, *M* (*SD*)	3.1 (1.8)	3.1 (1.9)	3.1 (1.8)	3.0 (1.8)	3.0 (1.8)	0.096
Relationship status, *n* (%)						< 0.001
Single	328 (3.0)	77 (2.7)	75 (2.6)	84 (3.2)	92 (3.3)	
Dating/engaged	157 (1.4)	33 (1.2)	39 (1.3)	33 (1.3)	52 (1.9)	
Partner	1715 (15.5)	419 (15.0)	417 (14.3)	399 (15.4)	480 (17.3)	
Married	6649 (60.0)	1719 (61.3)	1845 (63.3)	1583 (61.0)	1502 (54.2)	
Divorced/separated	2180 (19.7)	536 (19.1)	526 (18.0)	485 (18.7)	633 (22.8)	
Other	22 (0.2)	5 (0.2)	6 (0.2)	6 (0.2)	5 (0.2)	
Number of children, *M* (*SD*)	1.8 (1.5)	2.0 (1.5)	1.9 (1.5)	1.8 (1.5)	1.6 (1.5)	< 0.001
Big Five personality traits, *n* (%)						
Openness	4.9 (1.1)	4.9 (1.1)	4.9 (1.1)	4.9 (1.1)	5.1 (1.1)	< 0.001
Neuroticism	3.4 (1.1)	3.4 (1.1)	3.3 (1.1)	3.4 (1.2)	3.5 (1.2)	< 0.001
Extraversion	3.8 (1.2)	3.8 (1.2)	3.9 (1.1)	3.8 (1.1)	3.8 (1.2)	0.883
Conscientiousness	5.2 (1.0)	5.3 (1.0)	5.2 (1.0)	5.1 (1.0)	5.0 (1.1)	< 0.001
Agreeableness	1.7 (4.4)	5.4 (0.9)	5.4 (0.9)	5.4 (0.9)	5.3 (1.0)	0.135
Locus of health control, *M* (*SD*)	5.0 (1.1)	5.1 (1.1)	5.1 (1.1)	5.0 (1.1)	4.9 (1.1)	< 0.001
Political orientation, *M* (*SD*)	3.6 (1.4)	3.7 (1.4)	3.6 (1.4)	3.6 (1.3)	3.4 (1.4)	< 0.001
Hours putting on cosmetics, *M* (*SD*)	0.8 (2.7)	0.6 (2.7)	0.7 (2.1)	0.8 (2.8)	0.9 (3.1)	0.005
Hours commuting, *M* (*SD*)	5.2 (6.4)	4.8 (6.2)	5.2 (5.7)	5.4 (6.5)	5.5 (7.0)	0.001
Hours taking care of children, *M* (*SD*)	12.3 (29.5)	13.8 (31.2)	12.8 (29.8)	10.4 (27.0)	11.9 (29.7)	< 0.001
Hours doing housework, *M* (*SD*)	10.5 (9.3)	10.1 (9.8)	10.4 (8.8)	10.8 (9.2)	10.8 (9.2)	0.012
Hours working, *M* (*SD*)	26.9 (20.5)	28.1 (19.6)	27.3 (20.7)	25.9 (20.5)	26.1 (21.0)	< 0.001
**Psychological wellbeing**						
Life satisfaction, *M* (*SD*)	5.3 (1.2)	5.4 (1.1)	5.4 (1.1)	5.3 (1.2)	5.1 (1.3)	< 0.001
Self-steem, *M* (*SD*)	5.2 (1.2)	5.4 (1.2)	5.3 (1.2)	5.2 (1.2)	5.1 (1.3)	< 0.001
Body satisfaction, *M* (*SD*)	4.3 (1.7)	4.6 (1.6)	4.4 (1.6)	4.3 (1.6)	3.9 (1.7)	< 0.001
**Social wellbeing**						
Sense of community, *M* (*SD*)	5.1 (1.0)	4.9 (1.4)	4.8 (1.4)	4.8 (1.4)	4.5 (1.6)	< 0.001
Sense of belonging, *M* (*SD*)	6.0 (1.1)	5.1 (1.0)	5.2 (1.0)	5.1 (1.0)	4.9 (1.1)	< 0.001
Social support, *M* (*SD*)	5.4 (1.0)	6.0 (1.0)	6.1 (1.0)	6.0 (1.0)	5.9 (1.1)	0.072
**Social factors**						
Cyberbullying, ever, *n* (%)						< 0.001
No	9419 (85.0)	2399 (85.6)	2528 (86.7)	2269 (87.4)	2223 (80.2)	
Yes	1392 (12.6)	310 (11.1)	324 (11.1)	274 (10.6)	484 (17.5)	
Engaged/married, *n* (%)						0.034
No	10,932 (98.6)	2770 (98.9)	2877 (98.7)	2567 (98.8)	2718 (98.1)	
Yes	112 (1.0)	21 (0.7)	27 (0.9)	23 (0.9)	41 (1.5)	
Divorced/separated, *n* (%)						0.012
No	10,970 (99.0)	2761 (98.5)	2892 (99.2)	2577 (99.2)	2740 (98.9)	
Yes	74 (0.7)	30 (1.1)	12 (0.4)	13 (0.5)	19 (0.7)	
Harassed/assaulted/sexually assaulted, *n* (%)						0.242
No	11,031 (99.5)	2787 (99.5)	2901 (99.5)	2590 (99.7)	2753 (99.4)	
Yes	54 (0.5)	15 (0.5)	14 (0.5)	7 (0.3)	18 (0.6)	
**Character strengths and prosocial behavior**						
Volunteering (h/week), *M* (*SD*)	1.7 (4.4)	1.9 (4.9)	1.7 (3.9)	1.7 (4.4)	1.7 (4.4)	0.001
Charity donations ($NZ), *M* (*SD*)	868.6 (3323.6)	1011.7 (3919.5)	969.4 (3729.7)	797.9 (2857.0)	685.7 (2523.1)	< 0.001
Self-control	4.4 (1.3)	4.6 (1.3)	4.5 (1.3)	4.5 (1.3)	4.2 (1.4)	< 0.001
**Physical health**						
Subjective health, *M* (*SD*)	5.1 (1.1)	5.2 (1.1)	5.2 (1.1)	5.1 (1.1)	4.9 (1.2)	< 0.001
Sleep (h/day), *M* (*SD*)	6.9 (1.1)	7.0 (1.2)	6.9 (1.1)	7.0 (1.1)	6.8 (1.2)	< 0.001
Body mass index (kg/m^2^), *M* (*SD*)	27.3 (5.8)	26.3 (5.3)	27.0 (5.5)	27.5 (5.5)	28.6 (6.4)	< 0.001
Circulatory system diseases, *n* (%)						< 0.001
No	8502 (76.7)	2261 (80.7)	2233 (76.6)	1985 (76.4)	2023 (73.0)	
Yes	2583 (23.3)	541 (19.3)	682 (23.4)	612 (23.6)	748 (27.0)	
Musculoskeletal system diseases, *n* (%)						0.546
No	10378 (93.6)	2637 (94.1)	2731 (93.7)	2428 (93.5)	2582 (93.2)	
Yes	707 (6.4)	165 (5.9)	184 (6.3)	169 (6.5)	189 (6.8)	
Consequences of external causes, *n* (%)						0.151
No	10,817 (97.6)	2748 (98.1)	2833 (97.2)	2537 (97.7)	2699 (97.4)	
Yes	268 (2.4)	54 (1.9)	82 (2.8)	60 (2.3)	72 (2.6)	
**Mental health**						
Psychological distress, *M* (*SD*)	0.8 (0.6)	0.8 (0.6)	0.8 (0.6)	0.8 (0.6)	0.9 (0.7)	< 0.001
Mental disorder, *n* (%)						< 0.001
No	9200 (83.0)	2364 (84.4)	2453 (84.2)	2177 (83.8)	2206 (79.6)	
Yes	1885 (17.0)	438 (15.6)	462 (15.8)	420 (16.2)	565 (20.4)	
**Health behavior**						
Alcohol use, *M* (*SD*)	1.3 (0.8)	1.2 (0.8)	1.3 (0.8)	1.3 (0.8)	1.3 (0.9)	< 0.001
Exercise (h/week), *M* (*SD*)	5.8 (7.7)	5.7 (7.9)	6.0 (7.9)	5.9 (7.1)	5.7 (7.9)	0.422
Smoking, ever *n* (%)						< 0.001
No	10283 (92.8)	2593 (92.5)	2734 (93.8)	2442 (94.0)	2514 (90.7)	
Yes	678 (6.1)	163 (5.8)	153 (5.2)	136 (5.2)	226 (8.2)	

^a^This table was created based on non-imputed data

^b^All variables in Table 1 were assessed in the pre-baseline wave (T_0_)

^c^The percentages in some sections may not add up to 100% due to rounding

^d^Include workers from the following categories: community and persona, labourers, machinery operators, sales workers, technicians and trade

^e^Other working categories not included in the classification above

The only variables for which there was little evidence of differences across the quartiles of total screen-based leisure time were income attribution, New Zealand socioeconomic index, extraversion, agreeableness, social support, weekly hours spent exercising, being assaulted or harassed, having a musculoskeletal disease, and having health consequences of external causes.

Participants in the highest quartile of total screen-based leisure time at T_1_ had higher body mass index (quartile 4 mean = 28.6 kg/m^2^, overall mean = 27.3 kg/m^2^), worse subjective health (quartile 4 mean = 4.9, overall mean = 5.1), donated less money to charity (quartile 4 mean = 685.7 $NZ, overall mean = 868.6 $NZ), reported less self-control (quartile 4 mean = 4.2, overall mean = 4.4), and had lower body satisfaction (quartile 4 mean = 3.9, overall mean = 4.3) at T_0_.

### Primary Analysis

In our multivariable analysis with total screen-based leisure time and time spent in each of the screen-based leisure activities as continuous variables (Table 2), our models detected very modest associations between time spent in screen-based leisure activities and the outcomes examined. Results for the associations of both total screen-based leisure time and time in each screen-based leisure activity with the outcomes are described below.

**Table 2:** Relationship between time spent in screen-based leisure activities (2017) and subsequent outcomes (2019) in the total sample

**Outcomes by theme**	**Total screen-based leisure time**	**Browsing the internet**	**Watching videos**	**Watching or reading news**	**Using social media**	**Playing video games**
	***β*/OR/RR (95%CI)**^**a,b,c**^	***β*/OR/RR (95%CI)**^**a,b,c**^	***β*/OR/RR (95%CI)**^**a,b,c**^	***β*/OR/RR (95%CI)**^**a,b,c**^	***β*/OR/RR (95%CI)**^**a,b,c**^	***β*/OR/RR (95%CI)**^**a,b,c**^
**Psychological wellbeing**						
Life satisfaction,	− 0.01 (− 0.03, 0.00)	− 0.00 (− 0.02, 0.01)	− 0.02 (− 0.04, − 0.00)*	− 0.01 (− 0.03, 0.00)	− 0.00 (− 0.02, 0.02)	− 0.01 (− 0.03, 0.00)
Self-esteem	0.00 (− 0.01, 0.02)	− 0.00 (− 0.02, 0.01)	0.00 (− 0.01, 0.02)	0.00 (− 0.01, 0.02)	0.01 (− 0.01, 0.03)	− 0.01 (− 0.03, 0.01)
Body satisfaction	− 0.03 (− 0.05, − 0.01)**	− 0.03 (− 0.04, − 0.01)*	− 0.01 (− 0.03, 0.01)	− 0.02 (− 0.03, − 0.00)*	− 0.02 (− 0.04, − 0.00)*	− 0.01 (− 0.03, 0.01)
**Social wellbeing**						
Sense of community	− 0.01 (− 0.03, 0.01)	− 0.02 (− 0.04, 0.00)	0.01 (− 0.01, 0.03)	− 0.00 (− 0.02, 0.01)	− 0.00 (− 0.02, 0.02)	− 0.01 (− 0.03, 0.01)
Sense of belonging	− 0.00 (− 0.02, 0.02)	− 0.01 (− 0.02, 0.01)	0.01 (− 0.01, 0.02)	− 0.00 (− 0.02, 0.01)	0.00 (− 0.02, 0.02)	− 0.00 (− 0.02, 0.01)
Social support	− 0.01 (− 0.02, 0.01)	− 0.01 (− 0.03, 0.01)	0.00 (− 0.01, 0.02)	− 0.02 (− 0.03, 0.00)	0.01 (− 0.01, 0.02)	0.01 (− 0.01, 0.03)
**Social factors**						
Recently cyberbullied	1.03 (0.89, 1.19)	1.04 (0.90, 1.19)	1.02 (0.87, 1.19)	0.93 (0.79, 1.09)	0.99 (0.85, 1.14)	1.13 (0.99, 1.30)
Engaged/married/entered a civil union^**d**^	0.97 (0.79, 1.20)	1.02 (0.84, 1.24)	0.82 (0.63, 1.06)	0.98 (0.75, 1.28)	1.00 (0.84, 1.18)	1.09 (0.92, 1.29)
Divorced/separated^**e**^	0.80 (0.60, 1.06)	0.95 (0.74, 1.22)	0.87 (0.65, 1.17)	0.75 (0.53, 1.05)	0.72 (0.51, 1.04)	0.85 (0.61, 1.18)
Assaulted/harassed/attacked	0.97 (0.85, 1.09)	0.98 (0.86, 1.11)	0.95 (0.83, 1.09)	0.97 (0.86, 1.10)	1.03 (0.93, 1.14)	0.96 (0.85, 1.08)
**Character strengths and prosocial behavior**						
Volunteering (h/week)	0.03 (0.00, 0.05)*	0.03 (0.01, 0.06)*	− 0.00 (− 0.02, 0.02)	0.01 (− 0.01, 0.03)	0.02 (− 0.01, 0.04)	− 0.01 (− 0.03, 0.02)
Charity donations ($NZ)	− 0.01 (− 0.03, 0.01)	− 0.00 (− 0.03, 0.02)	− 0.01 (− 0.03, 0.01)	− 0.01 (− 0.03, 0.01)	0.00 (− 0.02, 0.03)	− 0.01 (− 0.03, 0.02)
Self-control	− 0.03 (− 0.04, − 0.01)*	− 0.01 (− 0.03, 0.01)	− 0.03 (− 0.05, − 0.02)**	− 0.01 (− 0.03, 0.01)	− 0.01 (− 0.03, 0.00)	− 0.01 (− 0.03, 0.00)
**Physical health**						
Subjective health	− 0.03 (− 0.04, − 0.01)*	− 0.02 (− 0.03, 0.00)	− 0.02 (− 0.04, − 0.00)*	− 0.01 (− 0.03, 0.00)	− 0.00 (− 0.02, 0.02)	− 0.03 (− 0.04, − 0.01)*
Sleep hours (h/day)	− 0.03 (− 0.05, − 0.01)*	− 0.02 (− 0.04, − 0.00)*	− 0.03 (− 0.05, − 0.01)*	0.00 (− 0.02, 0.02)	− 0.01 (− 0.03, 0.01)	− 0.02 (− 0.04, − 0.00)*
Body mass index	0.03 (0.01, 0.04)**	0.01 (− 0.00, 0.02)	0.02 (0.01, 0.03)**	0.01 (− 0.00, 0.02)	0.02 (0.01, 0.03)**	0.02 (0.01, 0.03)**
Circulatory system diseases	1.04 (0.99, 1.09)	1.05 (1.00, 1.11)*	1.01 (0.96, 1.05)	1.00 (0.97, 1.04)	0.99 (0.94, 1.05)	1.03 (0.98, 1.08)
Musculoskeletal system diseases	1.04 (0.93, 1.16)	0.97 (0.86, 1.09)	1.09 (0.99, 1.21)	1.09 (1.00, 1.19)	0.94 (0.83, 1.06)	1.00 (0.90, 1.11)
Consequences of external causes	1.09 (0.94, 1.27)	1.05 (0.90, 1.23)	1.04 (0.89, 1.20)	1.08 (0.97, 1.20)	1.08 (0.94, 1.23)	0.98 (0.83, 1.14)
**Mental health**						
Psychological distress	− 0.00 (− 0.02, 0.02)	− 0.00 (− 0.02, 0.02)	− 0.01 (− 0.03, 0.01)	0.01 (− 0.01, 0.02)	0.00 (− 0.01, 0.02)	0.01 (− 0.01, 0.02)
Mental disorders	0.97 (0.92, 1.03)	0.96 (0.91, 1.02)	1.00 (0.95, 1.05)	0.99 (0.94, 1.04)	0.99 (0.95, 1.04)	1.01 (0.96, 1.05)
**Health behavior**						
Alcohol use	0.01 (− 0.00, 0.03)	0.01 (− 0.01, 0.02)	0.02 (0.00, 0.03)*	0.00 (− 0.01, 0.01)	0.01 (− 0.00, 0.03)	− 0.00 (− 0.02, 0.01)
Current smoking	1.06 (0.95, 1.17)	1.01 (0.90, 1.13)	1.13 (1.02, 1.25)*	1.02 (0.92, 1.13)	1.01 (0.91, 1.11)	0.99 (0.90, 1.09)
Exercise (h/week)	0.04 (0.01, 0.06)*	0.02 (− 0.00, 0.04)	0.03 (0.01, 0.05)*	0.03 (0.01, 0.05)*	0.03 (0.00, 0.05)*	0.01 (− 0.01, 0.03)

*OR* odds ratio, *CI* confidence interval

*p < 0.05 before Bonferroni correction; **p < 0.05 after Bonferroni correction (the *p*-value cut-off for Bonferroni correction is *p* = 0.05/24 outcomes = *p* < 0.002)

^a^All continuous outcomes (life satisfaction, self-esteem, sense of community, sense of belonging, social support, volunteering hours, amount of charity donations, self-control, subjective health, psychological distress, hours of sleep, body mass index, body satisfaction, alcohol intake, hours of exercise) were standardized (mean = 0, standard deviation = 1), and the standardized *β* was calculated through linear regression. Standard deviations for all continuous outcomes can be seen in Table S2. For binary outcomes with a prevalence < 10%, logistical regression models were run to calculate odds ratios (been cyberbullied, engaged, married or entered a civil union, divorced or separated, current smoking, diagnose of musculoskeletal system diseases, and health consequences of external causes outcomes). For binary outcomes with a prevalence > 10% (diagnose of mental health or circulatory system diseases), generalized linear models with a log link and Poisson distribution were run to calculate relative risks

^b^All models controlled for pre-baseline covariates (age, gender, ethnicity, sexual orientation, been born in New Zealand, resident in urban area, New Zealand socioeconomic index, occupation, income attribution, educational level, religion, relationship status, political orientation, openness, neuroticism, extraversion, conscientiousness, agreeableness, health locus of control, number of children, hours putting on cosmetics hours commuting, hours looking after children, hours of housework, hours working, pre-baseline screen time activities, and pre-baseline levels for all outcomes)

^c^Regression was performed by using the multiple imputed sample (*n* = 11,085)

^d^For this outcome, only participants married at pre-baseline were analyzed (*n* = 6649)

^e^For this outcome, only participants that were not married at pre-baseline were analyzed (*n* = 4436)

#### Associations Between Total Screen-Based Leisure Time and Outcomes

A standard difference increment in total screen-based leisure time (SD = 24.3 weekly hours) was associated with a very modest increase in body mass index (beta, 0.03; 95%CI, 0.01, 0.04) and with a very modest decrease in body satisfaction (beta, − 0.03; 95%CI, − 0.05, − 0.01), even after Bonferroni correction. Evidence of possible associations was also found between total screen-based leisure time and increases in number of hours spent exercising each week (beta, 0.04; 95%CI, 0.01, 0.06) and volunteering each week (beta, 0.03; 95%CI, 0.00, 0.05), as well as decreases in average daily hours of sleep (beta, − 0.03; 95%CI, − 0.05, − 0.01), self-control (beta, − 0.03; 95%CI, − 0.05, − 0.01), and subjective health (beta, − 0.03; 95%CI, − 0.04, − 0.01). However, none of these associations passed the Bonferroni-corrected threshold.

#### Associations Between Each Screen-Based Activity and Outcomes

Time spent browsing the internet evidenced possible associations with an increased risk of circulatory system diseases (odds ratio (OR), 1.05; 95%CI, 1.00, 1.11), an increase in number of hours spent volunteering each week (beta, 0.03; 95%CI, 0.01, 0.06), a decrease of average hours spent sleeping each day (beta, − 0.02; 95%CI, − 0.04, − 0.00), and a decrease in body satisfaction (beta, − 0.03; 95%CI, − 0.04, − 0.01), although none of these associations passed the Bonferroni-corrected threshold.

Time spent watching videos was associated with a decrease in self-control (beta, − 0.03; 95%CI, − 0.05, − 0.02) and an increase in body mass index (beta, 0.02; 95%CI, 0.01, 0.03), even after Bonferroni correction. There was also possible evidence of associations with decreases in life satisfaction (beta, − 0.02; 95%CI, − 0.04, − 0.00), average hours spent sleeping each day (beta, − 0.03; 95%CI, − 0.05, − 0.01), and subjective health (beta, − 0.02; 95%CI, − 0.04, − 0.00), as well as increases in alcohol use (beta, 0.02; 95%CI, 0.00, 0.03), smoking behavior (OR, 1.13; 95%CI, 1.02, 1.25), and number of hours spent exercising each week (beta, 0.03; 95%CI, 0.01, 0.05). However, none of these associations passed the Bonferroni-corrected threshold.

Similarly, time spent reading or watching news evidenced possible associations with an increase in number of hours spent exercising each week (beta, 0.03; 95%CI, 0.01, 0.05) and a decrease in body satisfaction (beta, − 0.02; 95%CI, − 0.03, − 0.00), although neither passed the Bonferroni-corrected threshold.

Time spent on social media was associated with an increase in body mass index (beta, 0.02; 95%CI, 0.01, 0.03), even after Bonferroni correction. Possible associations were also found with an increase in number of hours spent exercising each week (beta, 0.03; 95%CI, 0.00, 0.05) and a decrease in body satisfaction (beta, − 0.02; 95%CI, − 0.04, − 0.00), although both of these associations failed to pass the Bonferroni-corrected threshold.

Finally, time spent playing video games was associated with an increase in body mass index (beta, 0.02; 95%CI, 0.01, 0.03), even after Bonferroni correction. Evidence of possible associations was also found with decreases in subjective health (beta, − 0.03; 95%CI, − 0.04, − 0.01) and average daily hours of sleep (beta, − 0.02; 95%CI, − 0.04, − 0.00), neither of which passed the Bonferroni-corrected threshold.

### Secondary Analysis

After stratifying the sample into two age groups (< 40 years vs. ≥ 40 years), associations tended to be quite similar across the two groups but there were some differences (Tables 3 and 4). The most noticeable differences between the age groups are described below.

**Table 3:** Relationship between time spent in screen-based leisure activities (2017) and subsequent outcomes (2019) in the subsample < 40 years old

**Outcomes by theme**	**Total screen-based leisure time**	**Browsing the internet**	**Watching videos**	**Watching or reading news**	**Using social media**	**Playing video games**
	***β*/OR/RR (95%CI)**^**a,b,c**^	***β*/OR/RR (95%CI)**^**a,b,c**^	***β*/OR/RR (95%CI)**^**a,b**^	***β*/OR/RR (95%CI)**^**a,b,c**^	***β*/OR/RR (95%CI)**^**a,b,c**^	***β*/OR/RR (95%CI)**^**a,b,c**^
**Psychological wellbeing**						
Life satisfaction	− 0.05 (− 0.08, − 0.01)*	− 0.02 (− 0.05, 0.01)	− 0.04 (− 0.09, − 0.00)*	− 0.04 (− 0.09, 0.01)	− 0.02 (− 0.05, 0.00)	− 0.05 (− 0.08, − 0.02)**
Self-esteem	− 0.01 (− 0.04, 0.03)	− 0.01 (− 0.04, 0.02)	0.01 (− 0.04, 0.05)	0.00 (− 0.04, 0.05)	0.01 (− 0.02, 0.04)	− 0.03 (− 0.07, − 0.00)*
Body satisfaction	− 0.01 (− 0.05, 0.02)	− 0.01 (− 0.04, 0.03)	− 0.01 (− 0.05, 0.03)	0.01 (− 0.03, 0.06)	− 0.00 (− 0.03, 0.02)	− 0.03 (− 0.06, 0.00)
**Social wellbeing**						
Sense of community	− 0.02 (− 0.06, 0.02)	− 0.03 (− 0.07, 0.01)	0.02 (− 0.03, 0.07)	− 0.04 (− 0.09, 0.01)	− 0.00 (− 0.03, 0.03)	− 0.02 (− 0.05, 0.02)
Sense of belonging	− 0.03 (− 0.07, − 0.00)*	− 0.02 (− 0.05, 0.01)	− 0.05 (− 0.09, − 0.01)*	− 0.05 (− 0.09, − 0.01)*	0.00 (− 0.02, 0.03)	− 0.02 (− 0.05, 0.01)
Social support	0.00 (− 0.03, 0.03)	0.00 (− 0.03, 0.03)	− 0.01 (− 0.05, 0.03)	− 0.03 (− 0.08, 0.02)	0.01 (− 0.02, 0.04)	0.00 (− 0.03, 0.03)
**Social factors**						
Recently cyberbullied	1.06 (0.80, 1.41)	1.10 (0.84, 1.45)	1.02 (0.73, 1.43)	0.83 (0.51, 1.37)	0.88 (0.61, 1.26)	1.26 (0.96, 1.66)
Engaged/married/entered a civil union^**d**^	0.96 (0.73, 1.25)	0.99 (0.78, 1.26)	0.75 (0.50, 1.13)	0.97 (0.66, 1.41)	1.03 (0.84, 1.27)	1.05 (0.81, 1.35)
Divorced/separated^**e**^	0.90 (0.41, 1.98)	0.86 (0.42, 1.78)	0.77 (0.54, 1.08)	0.95 (0.40, 2.22)	0.64 (0.29, 1.43)	0.84 (0.33, 2.16)
Assaulted/harassed/attacked	0.94 (0.76, 1.17)	1.00 (0.81, 1.23)	0.80 (0.59, 1.08)	1.00 (0.76, 1.31)	0.99 (0.83, 1.17)	0.99 (0.81, 1.21)
**Character strengths and prosocial behavior**						
Volunteering (h/week)	0.00 (− 0.03, 0.03)	0.01 (− 0.02, 0.04)	− 0.01 (− 0.05, 0.02)	− 0.02 (− 0.07, 0.02)	0.02 (− 0.01, 0.04)	− 0.02 (− 0.05, 0.01)
Charity donations ($NZ)	0.00 (− 0.01, 0.02)	0.01 (− 0.01, 0.02)	− 0.00 (− 0.02, 0.02)	− 0.01 (− 0.03, 0.02)	0.00 (− 0.01, 0.02)	0.00 (− 0.01, 0.02)
Self-control	− 0.02 (− 0.05, 0.01)	0.00 (− 0.03, 0.03)	− 0.03 (− 0.07, 0.01)	− 0.03 (− 0.07, 0.02)	− 0.02 (− 0.05, 0.00)	− 0.00 (− 0.03, 0.03)
**Physical health**						
Subjective health	− 0.02 (− 0.06, 0.01)	− 0.01 (− 0.04, 0.02)	− 0.02 (− 0.06, 0.02)	− 0.01 (− 0.06, 0.03)	− 0.00 (− 0.03, 0.02)	− 0.04 (− 0.07, − 0.01)*
Sleep hours (h/day)	− 0.02 (− 0.06, 0.01)	− 0.02 (− 0.05, 0.02)	− 0.04 (− 0.09, 0.01)	0.04 (− 0.01, 0.09)	− 0.01 (− 0.04, 0.02)	− 0.01 (− 0.04, 0.03)
Body mass index	0.04 (0.02, 0.06)**	0.02 (0.00, 0.04)*	0.04 (0.01, 0.06)*	0.01 (− 0.02, 0.04)	0.02 (0.01, 0.04)*	0.02 (0.00, 0.04)*
Circulatory system diseases	1.22 (1.03, 1.44)*	1.15 (0.97, 1.36)	1.29 (1.07, 1.55)*	1.07 (0.83, 1.38)	1.05 (0.90, 1.22)	1.09 (0.90, 1.32)
Musculoskeletal system diseases	0.79 (0.49, 1.27)	0.92 (0.61, 1.39)	1.00 (0.59, 1.72)	1.38 (0.92, 2.08)	0.69 (0.43, 1.10)	0.61 (0.36, 1.03)
Consequences of external causes	1.02 (0.71, 1.48)	0.99 (0.68, 1.45)	1.14 (0.76, 1.72)	0.90 (0.48, 1.69)	1.05 (0.79, 1.39)	0.78 (0.46, 1.32)
**Mental health**						
Psychological distress	0.02 (− 0.01, 0.06)	0.01 (− 0.03, 0.04)	0.01 (− 0.03, 0.05)	0.04 (− 0.01, 0.09)	0.02 (− 0.01, 0.05)	0.02 (− 0.02, 0.05)
Mental disorders	0.96 (0.88, 1.04)	0.97 (0.89, 1.06)	0.97 (0.88, 1.07)	0.94 (0.82, 1.07)	0.97 (0.91, 1.04)	1.00 (0.93, 1.07)
**Health behavior**						
Alcohol use	− 0.01 (− 0.04, 0.03)	− 0.02 (− 0.05, 0.01)	0.04 (0.00, 0.08)*	− 0.04 (− 0.08, 0.00)	0.01 (− 0.02, 0.03)	0.00 (− 0.03, 0.03)
Current smoking	1.09 (0.87, 1.36)	1.01 (0.80, 1.27)	1.21 (0.93, 1.58)	1.23 (0.89, 1.70)	1.10 (0.91, 1.32)	0.86 (0.67, 1.10)
Exercise (h/week)	0.04 (0.00, 0.07)	0.03 (− 0.01, 0.06)	0.01 (− 0.04, 0.05)	− 0.01 (− 0.06, 0.04)	0.03 (− 0.00, 0.06)	0.05 (0.01, 0.08)*

*OR* odds ratio, *CI* confidence interval

*p < 0.05 before Bonferroni correction; **p < 0.05 after Bonferroni correction (the *p*-value cut-off for Bonferroni correction is *p* = 0.05/24 outcomes = *p* < 0.002)

^a^All continuous outcomes (life satisfaction, self-esteem, sense of community, sense of belonging, social support, volunteering hours, amount of charity donations, self-control, subjective health, psychological distress, hours of sleep, body mass index, body satisfaction, alcohol intake, hours of exercise) were standardized (mean = 0, standard deviation = 1), and the standardized *β* was calculated through linear regression. Standard deviations for all continuous outcomes can be seen in Table S2. For binary outcomes with a prevalence < 10%, logistical regression models were run to calculate odds ratios (been cyberbullied, engaged, married or entered a civil union, divorced or separated, current smoking, diagnose of musculoskeletal or connective system diseases, and health consequences of external causes outcomes). For binary outcomes with a prevalence > 10% (diagnose of mental health or circulatory system diseases), generalized linear models with a log link and Poisson distribution were run to calculate relative risks

^b^All models controlled for pre-baseline covariates (age, gender, ethnicity, sexual orientation, been born in New Zealand, resident in urban area, New Zealand socioeconomic index, occupation, income attribution, educational level, religion, relationship status, political orientation, openness, neuroticism, extraversion, conscientiousness, agreeableness, health locus of control, number of children, hours putting on cosmetics hours commuting, hours looking after children, hours of housework, hours working, pre-baseline screen time activities, and pre-baseline levels for all outcomes)

^c^Regression was performed by using the multiple imputed sample of participants under 40 years old (*n* = 2296)

^d^For this outcome, only participants married at pre-baseline were analyzed (*n* = 968)

^e^For this outcome, only participants that were not married at pre-baseline were analyzed (*n* = 1328)

**Table 4:** Relationship between time spent in screen-based leisure activities (2017) and subsequent outcomes (2019) in the subsample ≥ 40 years old

Outcomes by theme	Total screen-based leisure time	Browsing the internet	Watching videos	Watching or reading news	Using social media	Playing video games
	***β*/OR/RR (95%CI)**^**a,b,c**^	***β*/OR/RR (95%CI)**^**a,b,c**^	***β*/OR/RR (95%CI)**^**a,b,c**^	***β*/OR/RR (95%CI)**^**a,b,c**^	***β*/OR/RR (95%CI)**^**a,b,c**^	***β*/OR/RR (95%CI)**^**a,b,c**^
**Psychological wellbeing**						
Life satisfaction	− 0.00 (− 0.02, 0.02)	0.00 (− 0.02, 0.02)	− 0.01 (− 0.03, 0.00)	− 0.01 (− 0.02, 0.01)	0.01 (− 0.01, 0.03)	0.01 (− 0.01, 0.03)
Self-esteem	0.01 (− 0.01, 0.03)	0.00 (− 0.02, 0.02)	0.01 (− 0.01, 0.02)	0.01 (− 0.01, 0.02)	0.01 (− 0.02, 0.03)	0.00 (− 0.01, 0.02)
Body satisfaction	− 0.04 (− 0.06, − 0.02)**	− 0.04 (− 0.06, − 0.01)**	− 0.01 (− 0.03, 0.01)	− 0.02 (− 0.04, − 0.00)*	− 0.04 (− 0.06, − 0.01)*	0.00 (− 0.02, 0.02)
**Social wellbeing**						
Sense of community	− 0.01 (− 0.03, 0.02)	− 0.01 (− 0.03, 0.01)	0.00 (− 0.02, 0.02)	0.00 (− 0.02, 0.02)	− 0.01 (− 0.03, 0.02)	− 0.01 (− 0.03, 0.01)
Sense of belonging	0.01 (− 0.01, 0.03)	0.00 (− 0.02, 0.02)	0.02 (0.00, 0.04)*	0.00 (− 0.02, 0.02)	− 0.01 (− 0.03, 0.02)	0.01 (− 0.01, 0.03)
Social support	− 0.01 (− 0.03, 0.01)	− 0.02 (− 0.04, 0.00)	0.01 (− 0.01, 0.03)	− 0.01 (− 0.03, 0.00)	− 0.00 (− 0.03, 0.02)	0.01 (− 0.01, 0.03)
**Social factors**						
Recently cyberbullied	1.10 (0.92, 1.32)	1.09 (0.92, 1.29)	1.03 (0.86, 1.24)	0.97 (0.82, 1.14)	1.14 (0.95, 1.36)	1.08 (0.90, 1.30)
Engaged/married/entered a civil union^**d**^	1.03 (0.67, 1.58)	1.11 (0.73, 1.70)	0.83 (0.54, 1.28)	0.95 (0.58, 1.57)	1.05 (0.64, 1.72)	1.10 (0.87, 1.39)
Divorced/separated^**e**^	0.75 (0.53, 1.07)	0.98 (0.73, 1.32)	0.77 (0.54, 1.08)	0.74 (0.50, 1.08)	0.64 (0.39, 1.05)	0.85 (0.58, 1.26)
Assaulted/harassed/attacked	1.03 (0.87, 1.21)	0.99 (0.84, 1.17)	1.06 (0.90, 1.23)	0.98 (0.85, 1.13)	1.08 (0.94, 1.26)	0.96 (0.82, 1.13)
**Character strengths and prosocial behavior**						
Volunteering (h/week)	0.04 (0.01, 0.07)*	0.05 (0.02, 0.08)**	0.01 (− 0.02, 0.03)	0.02 (− 0.01, 0.04)	0.02 (− 0.02, 0.05)	− 0.00 (− 0.03, 0.03)
Charity donations ($NZ)	− 0.02 (− 0.05, 0.01)	− 0.01 (− 0.04, 0.02)	− 0.01 (− 0.04, 0.01)	− 0.01 (− 0.03, 0.02)	0.00 (− 0.03, 0.04)	− 0.01 (− 0.04, 0.02)
Self-control	− 0.03 (− 0.05, − 0.01)*	− 0.01 (− 0.03, 0.01)	− 0.03 (− 0.05, − 0.01)**	− 0.01 (− 0.02, 0.01)	− 0.01 (− 0.03, 0.01)	− 0.01 (− 0.03, 0.01)
**Physical health**						
Subjective health	− 0.03 (− 0.05, − 0.00)*	− 0.02 (− 0.04, 0.00)	− 0.02 (− 0.04, 0.00)	− 0.01 (− 0.03, 0.01)	− 0.00 (− 0.03, 0.02)	− 0.02 (− 0.04, − 0.00)
Sleep hours (h/day)	− 0.03 (− 0.06, − 0.01)*	− 0.03 (− 0.05, − 0.00)*	− 0.02 (− 0.04, 0.00)	− 0.00 (− 0.02, 0.02)	− 0.01 (− 0.03, 0.02)	− 0.03 (− 0.06, − 0.01)*
Body mass index	0.02 (0.01, 0.03)*	− 0.00 (− 0.01, 0.01)	0.02 (0.00, 0.03)*	0.01 (− 0.00, 0.02)	0.02 (0.00, 0.03)*	0.03 (0.01, 0.04)**
Circulatory system diseases	1.03 (0.97, 1.08)	1.05 (0.99, 1.10)	0.99 (0.95, 1.04)	1.00 (0.96, 1.04)	1.00 (0.94, 1.07)	1.03 (0.98, 1.08)
Musculoskeletal system diseases	1.09 (0.97, 1.23)	1.00 (0.88, 1.13)	1.12 (1.00, 1.24)*	1.09 (1.00, 1.19)	0.98 (0.85, 1.14)	1.05 (0.94, 1.17)
Consequences of external causes	1.12 (0.93, 1.33)	1.08 (0.89, 1.30)	1.03 (0.87, 1.22)	1.10 (0.98, 1.23)	1.07 (0.91, 1.27)	1.00 (0.84, 1.19)
**Mental health**						
Psychological distress	− 0.01 (− 0.04, 0.01)	− 0.01 (− 0.03, 0.01)	− 0.02 (− 0.04, − 0.00)	− 0.00 (− 0.02, 0.02)	− 0.01 (− 0.03, 0.02)	0.00 (− 0.02, 0.02)
Mental disorders	0.99 (0.93, 1.06)	0.97 (0.90, 1.04)	1.01 (0.95, 1.08)	1.00 (0.94, 1.05)	1.02 (0.96, 1.08)	1.01 (0.94, 1.08)
**Health behavior**						
Alcohol use	0.03 (0.01, 0.04)*	0.02 (0.00, 0.04)*	0.01 (− 0.00, 0.03)	0.01 (− 0.00, 0.02)	0.02 (0.00, 0.04)	− 0.00 (− 0.02, 0.01)
Current smoking	1.04 (0.91, 1.18)	1.00 (0.86, 1.15)	1.10 (0.98, 1.24)	1.00 (0.89, 1.12)	0.94 (0.82, 1.09)	1.01 (0.90, 1.12)
Exercise (h/week)	0.04 (0.01, 0.07)*	0.01 (− 0.02, 0.04)	0.04 (0.01, 0.06)	0.04 (0.01, 0.06)*	0.02 (− 0.01, 0.05)	− 0.00 (− 0.03, 0.02)

*OR* odds ratio, *CI* confidence interval

*p < 0.05 before Bonferroni correction; **p < 0.05 after Bonferroni correction (the *p*-value cut-off for Bonferroni correction is *p* = 0.05/24 outcomes = *p* < 0.002)

^a^All continuous outcomes (life satisfaction, self-esteem, sense of community, sense of belonging, social support, volunteering hours, amount of charity donations, self-control, subjective health, psychological distress, hours of sleep, body mass index, body satisfaction, alcohol intake, hours of exercise) were standardized (mean = 0, standard deviation = 1), and the standardized *β* was calculated through linear regression. Standard deviations for all continuous outcomes can be seen in Table S2. For binary outcomes with a prevalence < 10%, logistical regression models were run to calculate odds ratios (been cyberbullied, engaged, married or entered a civil union, divorced or separated, current smoking, diagnose of musculoskeletal or connective system diseases, and health consequences of external causes outcomes). For binary outcomes with a prevalence > 10% (diagnose of mental health or circulatory system diseases), generalized linear models with a log link and Poisson distribution were run to calculate relative risks

^b^All models controlled for pre-baseline covariates (age, gender, ethnicity, sexual orientation, been born in New Zealand, resident in urban area, New Zealand socioeconomic index, occupation, income attribution, educational level, religion, relationship status, political orientation, openness, neuroticism, extraversion, conscientiousness, agreeableness, health locus of control, number of children, hours putting on cosmetics hours commuting, hours looking after children, hours of housework, hours working, pre-baseline screen time activities, and pre-baseline levels for all outcomes)

^c^Regression was performed by using the multiple imputed sample of participants aged 40 years old or more (*n* = 8789)

^d^For this outcome, only participants married at pre-baseline were analyzed (*n* = 5681)

^e^For this outcome, only participants that were not married at pre-baseline were analyzed (*n* = 3108)

First, we found evidence of an association between total screen-based leisure time and browsing the internet with an increase in number of weekly hours spent volunteering was among older participants, but there was little evidence of an association among those in the younger group. This association passed the Bonferroni-corrected threshold for time spent browsing the internet, but not for total screen-based leisure time. Second, the older age group had slightly stronger associations between time spent in screen-based leisure activities and body satisfaction than the younger age group, which passed the Bonferroni-corrected threshold for total screen-based leisure time and time spent browsing the internet. On the contrary, the associations between time spent in screen-based leisure activities with life satisfaction and body mass index tended to be stronger in the younger age group. In this group, the associations of total screen-based leisure time with increased body mass index and of time playing video games with decreased life satisfaction passed the Bonferroni-corrected threshold. Finally, there was possible evidence that time spent in watching or reading news and time spent in watching videos were associated with a decrease in sense of belonging among the younger age group, whereas time watching videos among the older age group evidenced a possible association with an increased in sense of belonging; however, none of these associations passed the Bonferroni-corrected threshold.

### Sensitivity Analyses

Tables S3, S4, and S5 show the *E*-values for the robustness of the associations found in the total sample, the younger age group, and the older age group, respectively. Most of the *E*-values showed point estimates around 1.20 for all associations that passed the conventional *p* < 0.05 threshold, suggesting that relatively modest unmeasured confounder associations between total screen-based leisure time (or time spent in specific screen-based leisure activities) and the outcomes could suffice to explain away the effect estimates. For example, an unmeasured confounder that was associated with both total screen-based leisure time and body mass index by a risk ratio of 1.18-fold each, conditional on the measured covariates, could fully explain away the observed association. Even weaker unmeasured confounder associations could suffice to shift the confidence intervals to include the null.

In the multivariable analyses by quartiles of total screen-based leisure time, most of the associations observed in our primary analysis were replicated (Table S6). Compared with those in the lowest quartile of total screen-based leisure time, participants in the highest quartile had worse body satisfaction, self-control, worse subjective health, and higher body mass index, which were upheld even after Bonferroni correction. There was some evidence of an association between being in the highest quartile of total screen-based leisure time and greater sense of community, a higher number of weekly hours spent volunteering, and higher alcohol consumption, although these associations did not pass the Bonferroni-corrected threshold.

Similar results were obtained after replicating the quartile-based analysis in the younger age group (Table S7) and the older age group (Table S8), with stronger associations detected for subjective health and body satisfaction in the younger subsample and stronger associations for body mass index, body satisfaction, and self-control in the older subsample. However, these associations only passed the Bonferroni-corrected threshold in the older age group.

Finally, our results were similar when models additionally adjusted for survey response format used by participants, with negligible changes at centesimal level for a few outcomes in all models (data not shown). Results were also equally similar after removing those who completed the wave 11 survey after the New Zealand government implemented lockdown measures on 25 March 2020 (data not shown).

Among the primary, secondary, and sensitivity analyses, screen-based leisure time evidenced the most robust associations with body mass index and body satisfaction. Because of the very modest sizes, we calculated Bayes factors for total screen-based leisure time with both outcomes, which supported the alternative hypothesis in each case (BF_body mass index_ < 0.001, BF_body satisfaction_ = 0.042).

## Discussion

In this study, we examined the associations of screen-based leisure time with a wide range of outcomes in a representative sample of New Zealanders. After 2 years of follow-up, total screen-based leisure time was more consistently associated with worse subsequent body mass index and body satisfaction than other outcomes. However, the strength of these associations was very modest, which suggests that changes in those outcomes will tend to occur only after investing a considerable amount of time in screen-based leisure activities. We also found possible evidence of associations between total screen-based leisure time and variables in several domains, including psychological wellbeing, physical and mental health, health behaviors, character strength, prosocial behavior, and social relationships, although these associations either were not consistent across our models or did not pass the Bonferroni-corrected threshold. When time spent in each screen-based leisure activity was examined separately, the strength of each of their associations varied to some extent for certain outcomes.

### Screen-Based Leisure Time and Psychological Wellbeing

Total screen-based leisure time showed little evidence of association with life satisfaction or self-esteem in the total sample. Only time spent watching videos evidenced a possible association with a decrease in life satisfaction. This association was stronger in the younger age group, particularly for total screen-based leisure time, time spent watching videos, and time spent playing video games. One possible explanation for this pattern of findings is that the activities could replace other more fulfilling ones, especially in the context of problematic internet use. Problematic internet use, by definition, implies that the excess of time spent on internet activities presents as a severe obstacle for some dimensions of daily life, such as social relationships or professional development, which could decrease life satisfaction. In this paper, we have assessed the effects of time spent in screen-based leisure activities, but we are unable to determine if time spent in those activities may have been problematic in our sample [28].

Some studies have pointed to a positive relationship between internet use and life satisfaction in retired adults through improvements in social relationships [29]. In our age-stratified sensitivity analysis, total screen-based leisure time showed little evidence of association with life satisfaction in the older age group, perhaps because this group also included non-retired adults.

### Screen-Based Leisure Time, Social Wellbeing, and Social Factors

In our primary analysis, we found little evidence that total screen-based time is associated with subsequent indicators of social wellbeing. However, in our age-stratified analysis, watching videos and watching or reading news were associated with a decrease in sense of belonging among the younger age group. On the contrary, watching videos was associated with an increase in sense of belonging among the older age group. These contrasting findings could be explained by differences in the motivations that these age groups might have for engaging in screen-based leisure time activities. On the one hand, younger adults tend to be more interested in news events with a strong mobilizing character or emotionalizing events, which could cause them to feel disconnected from other people [30]. Indeed, some evidence suggests that excessive time spent watching videos could lead to isolation from one’s social network [31]. On the other hand, watching videos with loved ones (e.g., a spouse, children) can facilitate family communication and bonding, which may enhance a person’s sense of belonging [32]. We were unable to identify the contexts and motivations behind screen use in our data, and therefore further research is needed to explore the mechanisms through which screen-based leisure time shapes social wellbeing across different generations.

### Screen-Based Leisure Time, Character Strengths, and Prosocial Behavior

There was some evidence of an association between total screen-based leisure time and lower self-control. This evidence was stronger for watching videos, which is traditionally considered a passive screen-based activity because the user needs lower amounts of focus compared to other screen-based leisure activities (e.g., playing video games). Technological developments in the last decade have made it even easier for people to access and watch videos. Video-based platforms or streaming services are designed to continuously propose content to their customers, and they make it very easy for people to spend time watching stream of videos. Because of this, binge-watching videos has become an increasingly popular activity. Some people who engage in excessive binge-watching are at risk of developing a behavioral-like addiction, which could impact negatively on a person’s social wellbeing [33]. Also, binge-watching videos can lead to a sense of remorse when the time spent in this activity could have been used for other more productive goals. Thus, people who repetitively binge-watch videos may experience chronic feelings of shame and guilt that could degrade their perceived capacity to control their own behavior [34]. Although the limits of our data preclude the possibility of identifying whether and how frequently participants in this study engaged in binge-watching sessions, future research might consider building on our findings by evaluating the implications of binge-watching for health and wellbeing.

Our study found some weak evidence suggesting that total screen-based leisure time and time spent browsing the internet were associated with an increase in number of hours spent volunteering each week. This finding corresponds with evidence reported in a previous cross-sectional study of 27 European countries [35], which reported a positive association between daily internet use and volunteering that was stronger among adults who were older, had lower education, and resided in rural areas. Our age-stratified analysis mirrored this finding, in that there was evidence of an association between total screen-based leisure time and volunteering in the older but not the younger subsample. For some older people, perhaps those who are retired more specifically, the internet may a valuable tool for finding volunteering opportunities [36, 37].

### Screen-Based Leisure Time, Exercising, and Body-Related Outcomes

Time spent in screen-based activities was related to an increase in body mass index and a decrease in body satisfaction after 2 years of follow-up. Our findings resonate with those that have previously been reported in the literature [38–42]. The association found between total screen-based leisure time and body mass index was similar for most screen-based activities, which could be due to snacking behaviors that can occur during this type of activity [39, 42]. This increase in body mass index could partially explain the decreased body satisfaction. Besides, body satisfaction could be negatively affected by weight stigma in media [43].

In contrast to the findings for body mass index and body satisfaction, total screen-based leisure time was associated with increased exercise after 2 years of follow-up. A stronger association was found for watching or reading the news than for the other screen-based activities, especially in the older subsample. We have three possible explanations for this finding. First, because exposure to content that stigmatizes obese people has been shown to decrease the self-efficacy of overweight people to exercise [44], it may be that weight stigma is less prevalent in news sources than in other types of screen-based leisure activities that were measured in this study [43]. Second, some people who watched the news may have done so while exercising. People who watch television while exercising are more likely to report positive effects after a home workout than those without a television, leading to more consistent exercise in the long-term [45]. Third, people with high amounts of screen-based leisure time might engage in intense vigorous activity for short periods to compensate for their sedentary behavior [46].

### Screen-Based Leisure Time and Other Health Outcomes

Total screen-based leisure time was also associated with decreased average daily hours of sleep. Similar associations were found for time spent browsing the internet, watching videos, or playing video games. These activities were practiced for more hours than watching or reading the news or using social media. Prior research shows that exposure to the light emitted from screens for extended periods of time can disrupt the melatonin-based brain circuits of sleep induction. Moreover, the duration of video game sessions or binge-watching television shows can also compete against sleeping time [42].

There was also evidence of an association between total screen-based leisure time and decreased subjective health, which could be explained (in part) by associations that were observed with lower subsequent average daily hours of sleep and higher subsequent body mass index. Moreover, advertisements and other messaging related to products that may pose a risk to health, such as unhealthy snacks, alcohol, or tobacco, can lead to an increase in these behaviors and degrade health over time [47, 48]. In the empirical literature, watching videos is commonly associated with alcohol consumption in adolescents and emerging adults but not among older adults [49]. A similar finding was observed in the present study, such that watching videos was more strongly associated with alcohol consumption and being a smoker in the younger subsample than in the older subsample. One explanation for these findings is that the content portrayed as part of movies or in advertisements linked to videos that people watch may foster the development of normative beliefs towards alcohol consumption and smoking that influence decisions about using these substances.

Additionally, it has been hypothesized that the repetitive dopamine-inducting stimulus associated with binge behaviors resembles other addictive behaviors and can sensitize the brain to other binging behaviors [50]. Along these lines, one recent study showed that hypermethylation of genes connected to the dopaminergic system may be more prevalent among individuals with problematic internet use [51]. However, it is not clear whether this finding might apply to binge-watching as well [50], and we were not able to assess binge-watching or problematic internet use with our data.

### Other Outcome-Wide Studies in the Literature

It is important to contextualize our findings based on the size and direction of the associations that were observed. First, effect sizes tended to be very modest (e.g., standardized beta coefficients did not exceed 0.03 in our primary analysis). Similar findings emerged in the sensitivity analysis in which we compared the quartile with the highest hours spent on screen-based leisure activities with the lowest (e.g., all standardized coefficients were less than 0.10).

Second, most of the associations that passed the conventional *p* < 0.05 indicated that the effects of screen-based leisure time tend to be adverse. This pattern of findings contrasts another outcome-wide study on internet use among older people from Japan [52]. In that study, internet use had a weak association with lower functional disabilities, better relationships, greater participation in hobbies or sport groups, and a higher likelihood of visiting a physician to have a routine health screening. One potential explanation for differences in results between the two studies is differences in assessment of screen use. Our study assessed all screen-based activities (including browsing the internet) based on the self-reported number of weekly hours invested in each activity. In Nakagomi et al. [52], participants were asked how many days per week they checked email or the internet. This approach cannot distinguish between moderate daily use (e.g., half an hour per day) and high daily use (e.g., 12 h per day), so the adverse effects of excessive use may be more difficult to detect.

Moreover, the reference category that was used in Nakagomi et al. [52] was not using the internet at all. In contrast, our primary analysis used total screen-based leisure time as a continuous variable to analyze the effect of an increase in one standard deviation of total screen-based leisure time (*SD* = 24.3 weekly hours). Furthermore, in our quartile-based sensitivity analysis, the first quartile of total screen-based leisure time ranged between 0 and 18 h. Thus, our study assessed the effect of increasing the time spent on screen-based leisure activities rather than comparing the effect of any screen-based leisure time against no screen-based leisure time at all. It is possible that the positive effects of internet use reported in Nakagomi et al. [52] might be due to the change from not using communication technologies to using them to some extent. On the contrary, the adverse effects observed in the present study might be related to the increments of time spent on screen-based activities that were already part of the daily schedules.

On the other side of the age spectrum, one study evaluated the association between screen-based leisure time and several outcomes related to psychological wellbeing, character strengths, and prosocial behaviors in a large sample of American children and teenagers aged 2–17 years [53]. They found that moderate or high levels of screen-based leisure time were associated with lower self-control, curiosity, emotional stability, and an ability to finish tasks; worse relationships with caregivers, more difficulties making friends, higher rates of depression or anxiety diagnosis, and higher rates of taking medication due to psychological or behavioral issues. The absolute effects sizes of these associations ranged from around 0.1 to 0.5, with larger effect sizes in high schoolers and those young with more than 7 daily hours of screen-based leisure time when compared to those with 1 h of daily screen-based leisure time. These effect sizes are substantially larger than the effect sizes detected in our study. One potential reason for this is that Twenge and Campbell’s [52] study used cross-sectional data. Effect sizes that are derived from cross-sectional data are not comparable with those derived from longitudinal data. Our multivariate analysis with longitudinal data underscores the unique contribution of screen-based leisure time to our list of outcomes after adjusting for other salient factors (e.g., pre-baseline values of the outcomes). Second, because our analyses control for pre-baseline screen-based leisure time, our models estimate the effect of screen-based leisure time *incidence* rather than *prevalence*. Third, our analyses evaluate the effects of screen-based leisure time after 2 years of follow-up instead of outcomes assessed contemporaneously; size effects corresponding with the latter approach could be stronger because the exposure and outcomes are assessed concurrently. Fourth, our findings are based on a sample of adults who might be less vulnerable to screen-based leisure time than children and adolescents. Still, despite differences in effect sizes, the findings of Twenge and Campbell [53] and the present study showed consistent associations between time spent in screen-based activities and lower self-control.

### Policy Implications

The weak findings in our study may still have important implications for health education and promotion. At the individual level, one person that reduces the amount of time spent on screen-based activities could have little favorable changes in several outcomes such as body mass index, body satisfaction, or other outcomes such as self-control and average daily hours of sleep. Despite the small effects of changes in screen-based leisure time, given the high prevalence of engaging in screen-based leisure within developed countries, aiming for a population-level reduction of time spent on this kind of leisure could be a valuable goal for national health promotion programs, especially when this type of behavioral change can be achieved at a very low cost. At the population level, even small decreases in risk factors can have substantial impacts on sanitary systems and the economy [54]. Our results showed the most robust effects of screen-based activities on body mass index. As obesity is a prevalent public health problem in developed and developing countries and is one of the leading causes of burden disease [55], health promotion program in countries where obesity prevalence is comparable to New Zealand would do well to consider and implement strategies to reduce unnecessary screen time.

### Strengths and Limitations

The present study has several limitations that ought to be considered. First, data about screen-based activities available in the NZAVS only addresses self-reported amount of time spent in such activities and not by the quality of the activities themselves. For example, we were not able to identify the devices used for certain activities, such as watching or reading news or playing video games, or whether participants engaged in those activities using an internet connection. In addition, messages that are portrayed in the media might contribute to shaping a person’s beliefs or attitudes, with downstream impacts on behavior. Hence, people could spend a similar amount of time exposed to media but encounter different kinds of messages because the content they watch or read might differ. Moreover, certain amounts of time spent engaged in screen-based activities could be perceived as “problematic” for some people, but not for others. For people with a tendency to engage in problematic internet use, gaming or binge-watching, the effects of time spent in screen-based activities on some outcomes could be stronger [56]. Additionally, because times spent engaged in the five screen-based activities were self-reported, those data may be subject to measurement error, and the effects of actual time spent engaged in screen-based activities may thus, in fact, likewise be larger than the estimates reported in this study. Longitudinal studies with objective measures of screen time (e.g., log-in records on technological devices) are needed to help address this limitation.

Second, we did not have data on other potential confounders that might be relevant to some of the outcomes we examined (e.g., trust in the internet, technological skills, problematic internet use). To better understand these limitations, we performed a sensitivity analysis using *E*-values to gauge how strong an unmeasured confounder would need to be associated with both screen-based leisure time and the outcome of interest to explain away their association (above and beyond the covariates already adjusted for in the analysis). In many cases, relatively modest unmeasured confounding would have been sufficient to explain away the associations. Importantly, however, we adjusted for a large number of relevant potential confounders, and any unmeasured confounders would have to confound associations between screen-based leisure time and the outcomes of interest in ways that are orthogonal to the confounders that we did include in the analytic models as covariates.

Third, due to changes in the NZAVS questionnaire from wave to wave, we limited to the follow-up period between the exposure of screen-based leisure time and the outcomes to 2 years because we wanted to maximize the number of relevant outcomes for inclusion in this study. Our results may have been more robust with an extended study period.

Fourth, our sample primarily consisted of older adults, with few emerging adults. The influence of time spent in screen-based leisure activities could be stronger among younger populations, in which case it would be important to replicate this study using data from younger samples.

Despite these limitations, key strengths of this study include the large sample size and the use of the outcome-wide analytic approach that allows for comparisons to be made across the outcomes while mitigating concerns about confounding and reverse causation. Furthermore, publishing both significant and null results from our outcome-wide analysis may be useful to future meta-analyses on this topic.

## Conclusion

After analyzing the association of time in screen-based activities with 24 outcomes related to health and wellbeing, we found some evidence that screen-based leisure time is related to several subsequent outcomes 2 years later. The most robust evidence of associations emerged with higher subsequent body mass index and lower subsequent body satisfaction, although effect sizes were very modest. Additional research is needed to determine whether our findings replicate in populations of different ages and over longer follow-up periods.

## Supplementary Information

Below is the link to the electronic supplementary material.

Supplementary file1 (DOCX 232 KB)

## Data Availability

The data used in this paper are part of theNZAVS. Full copies of the NZAVS data files are held by all members of the NZAVS management team and advisory board. A de-identified dataset containing the variables analyzed in this manuscript is available upon request from the corresponding author, or any member of the NZAVS advisory board for the purposes of replication or checking of any published study using NZAVS data. The Stata syntax used to test all models reported in this manuscript are available on the NZAVS OSF website: https://osf.io/75snb/.
