# Supplementary material for: Effects of Screen-Based Leisure Time on 24 Subsequent Health and Wellbeing Outcomes: A Longitudinal Outcome-Wide Analysis
Source: Int J Behav Med. 2024 Jul 18;32(6):919–38. doi: 10.1007/s12529-024-10307-0 (PMC12847154; doi:10.1007/s12529-024-10307-0)
Supplement: Supplementary file 1 — Supplementary file1 (DOCX 232 KB) [file 12529_2024_10307_MOESM1_ESM.docx]

Effects of screen-based leisure time on 24 subsequent health and wellbeing outcomes: A longitudinal outcome-wide analysis

**SUPPLEMENTAL MATERIAL**

**Table S1. List of variables**

| **Exposures**  **(Wave 2, 2017)** | **Variable Name in NZVAS** | | **Variable Type^1^** | | **Item Question** | |  |  |
| --- | --- | --- | --- | --- | --- | --- | --- | --- |
| *Primary analysis* | | | | |  | |  |  |
| Total screen-based leisure time | Sum of the 5 variables below | | Continuous (5 items, range: 0 to 417) | |  | |  |  |
| *Additional analysis* | | | | |  | |  |  |
| Hours/week playing video games | Hourscompgames | | Continuous (1 item, range: 0 to 100) | | 1. Hours… playing video/video games last week | |  |  |
| Hours/week browsing the internet | Hoursinternet | | Continuous (1 item, range: 0 to 140) | | 1. Hours… using the internet last week | |  |  |
| Hours/week using social media | Hourssocialmedia | | Continuous (1 item, range: 0 to 150) | | 1. Hours… using social media last week | |  |  |
| Hours/week watching/reading news | Hoursnews | | Continuous (1 item, range: 0 to 70) | | 1. Hours… watching/reading the news last week | |  |  |
| Hours/week watching videos | hourstv | | Continuous (1 item, range: 0 to 120) | | 1. Hours… watching TV/Netflix/Movies last week | |  |  |
| **Outcomes**  **(Wave 3, 2019)** | **Variable Name in NZVAS** | | **Variable Type** | | **Item Question** | |  |  |
| *Psychological wellbeing* | | | | |  | |  |  |
| Life satisfaction | lifesat | | Continuous (2 items, range: 1 to 7) | | *Satisfaction with life. Diener, E., Emmons, R. A., Larsen, R. J., & Griffin, S. (1985).*   1. I am satisfied with my life. 2. In most ways my life is close to ideal. | |  |  |
| Self-esteem | selfesteem | | Continuous (3 items, range: 1 to 7) | | *Rosenberg, M. (1965)*   1. On the whole am satisfied with myself. 2. Take a positive attitude toward myself. 3. Am inclined to feel that I am a failure. | |  |  |
| Body satisfaction | Bodysat | | Continuous (1 item, range: 1 to 7) | | 1. I am satisfied with the appearance, size and shape of my body. | |  |  |
| *Social wellbeing and social factors* | | | | |  | |  |  |
| Sense of community | swbsoc01 | | Continuous (1 item, range: 1 to 7) | | *Quality of Life 2008 Survey National Report (2009)*   1. I feel a sense of community with others in my local neighbourhood. | |  |  |
| Sense of belonging | belongn | | Continuous (3 items, range: 1 to 7) | | *Adapted From: Hagerty, B. M., & Patusky, K. (1995)*   1. Know that people in my life accept and value me. 2. Feel like an outsider. 3. Know that people around me share my attitudes and beliefs. | |  |  |
| Social support | 1support | | Continuous (2 items, range: 1 to 7) | | *Cutrona, C.E., & Russell, D.W. (1987).*   1. There are people I can depend on to help me if I really need it. 2. There is no one I can turn to for guidance in times of stress. 3. I know there are people I can turn to when I need help. | |  |  |
|  |  | |  | |  | |  |  |
| Recently cyberbullied^2^ | cyberbullyingrecent | | Binary (0 =No vs 1=Yes) | | 1. Has someone cyberbullied you in the last month? | |  |  |
| Engaged/married/entered a civil union | E18111 - Got married/entered a civil union  E18112 - Got remarried  E18113 - Got engaged engaged_married | | Binary (0 =No vs 1=Yes). Any participants who got married, engaged or remarried was scored as 1. | | 1. Got engaged, married or entered a civil union | |  |  |
| Divorced/separated | E12412 (divorced)  E12413 (Separated | | Binary (0 =No vs 1=Yes). Scored as 1 if either got divorced or separated | | 1. Got divorced or separated from your romantic partner/spouse | |  |  |
| Assaulted/harassed/attacked | E20211 (assaulted)  E20212 (harassed)  E20213 (sexually assaulted)  E20214 (Domestic violence) | | Binary (0 =No vs 1=Yes). Scored as 1 if ether got harassed, abused, secually harassed, or was a victim of domestic violence | | 1. E20211 - Someone assaulted you, abused you, or attacked you 2. E20212 - Someone sexually harrassed you 3. E20213 - Someone sexually assaulted you 4. E20214 - Domestic violence | |  |  |
| *Character strengths and prosocial behavior* | | | | |  | |  |  |
| Hours/week volunteering | Hourscharity | | Continuous (1 items, range: 0 to 70) | | 1. Hours spent … voluntary/charitable work last week | |  |  |
| Charity donations, $NZ | Charitydonate | | Continuous (1 item, range: 0 to 130000 $NZ) | | *Hoverd, W. J., & Sibley, C. G. (2010).*   1. How much money have you donated to charity in the last year? | |  |  |
| Self-control | Selfcontrol | | Continuous (2 items, range: 1 to 7) | | *Selfcontrol (Tangney, J. P., Baumeister, R. F., & Boone, A. L. (2004))*   1. In general, I have a lot of self-control. 2. I wish I had more self-discipline. | |  |  |
| *Physical health* | | | | |  | |  |  |
|  |  | |  | |  | |  |  |
| Subjective health | T11sfhealth | | Continuous (3 items, range: 1 to 7) | | *Short-Form Subjective Health Scale (General Health Perception Subscale)*  *Ware Jr, J. E., & Sherbourne, C.*  *D. (1992).*   1. In general, would you say your health is... 2. I seem to get sick a little easier than other people. 3. I expect my health to get worse. | |  |  |
| Sleep hours | hlthsleephours | | Continuous (1 item, range: 2.5 to 21) | | 1. During the past month, on average, how many hours of actual sleep did you get per night? | |  |  |
| Body mass index (kg/m^2^) | Hlthbmi | | Continuous (kg/m^2^  , range: 12.1 to 68.4) | | Kg/m^2^ | |  |  |
| Circulatory system diseases | Ch11_Circulatory.T02 | | Binary (0 =No vs 1=Yes) | | Diseases of the circulatory system | |  |  |
| Musculoskeletal system diseases | Ch15_Musculoskeletal.T02 | | Binary (0 =No vs 1=Yes) | | Diseases of the musculoskeletal system or connective tissue | |  |  |
| Consequences of external causes | Ch22_Injury.T02 | | Binary (0 =No vs 1=Yes) | | Injury, poisoning or certain other consequences of external causes | |  |  |
| *Mental health* | | | | | | |  | |
| Psychological distress | kessler6 | | Continuous (6 items, range: 0 to 4) | | *Kessler Psychological Distress Scale*. | |  |  |
| Mental disorders | Ch06_Mental.T02 | | Binary (0 =No vs 1=Yes) | | Mental, behavioural or neurodevelopmental disorders | |  |  |
| *Health behavior* | | | | | | |  |  |
| Alcohol use | alcoholfrequency  alcoholintensity | | Continuous (2 items, range: 0 to 4). | | *Babor, T. F., de la Fuente, J. R., Saunders, J., & Grant, M. (2001*)  *Doyle, S.R., Donovan, D.M., Kivlahan, D.R. (2007)*   1. How often do you have a drink containing alcohol? 2. How many drinks containing alcohol do you have on a typical day when drinking alcohol? | |  |  |
| Hours/week of exercise | hoursexercise | | Continuous (1 item, range: 0 to 100) | | 1. Hours spent … exercising/physical activity last week | |  |  |
| Current smoking^2^ | Smoker | | Binary (0 =No vs 1=Yes) | | 1. Do you currently smoke? | |  |  |
| **Covariates**  **(Wave 1, 2016)** | | **Variable Name in NZVAS** | | **Variable Type** | | **Item Question** | |  |
| Age (years) | | age | | Continuous (1 items, range: 17 to 98) | | 1. What is your date of birth? | |  |
| Gender | | gender | | Categorical (0= female 1=male 2=NA ) | | 1. What is your gender? (open-ended) | |  |
| Ethnicity | | ethniccatst09 | | Categorical (NZ European/Pakeha, Asian, Maori, Pacific) | | 1. Which ethnic group do you belong to (NZ census question)? | |  |
| Born in New Zealand | | bornnzt09 | | Binary (0 =No vs 1=Yes) | | 1. Which country were you born in? | |  |
| Urban area | | urbant09 | | Binary (0 =No vs 1=Yes 2=NA) | | 1. Urban/Rural distinction based on new 2018 Stats NZ Urban/Rural Indicator | |  |
| Sexual orientation | | sexual orientationl1t09 | | Cathegorical (0=Heterosexual 1=Homosexual 2=Other) | | 1. How would you describe your sexual orientation? (e.g., heterosexual, homosexual, straight, gay, lesbian, bisexual, etc.) | |  |
| Religion | | religion affiliation | | Cathegorical (0=No religion, 1= Christian, 2= Other ) | | 1. What religion or spiritual group? | |  |
| Educational level | | nzregaggregated | | Ordinal (No Qualification, Level1-3 Certificate, Level 5&6 Diploma, Level7 Bachelor Degree, Level 8&9 Postgraduate Degree, Level 10 Doctorate Degree , NA) | | 1. NZ Reg (0-10 education ordinal rank) | |  |
| Occupation | | occupationl1 | | Categorical (Clerical and administrative workers, Community and personal service workers, laborers, machinery operators and drivers, managers, professionals, residual/unreported, sales workers, technicians and trade workers ) | | 1. What is your current occupation? | |  |
| NZ socioeconomic index | | nzsei13t09 | | Continuous (1 items, range: 1 to 7) | | *Fahy, K. M., Lee, A., & Milne, B. J. (2017). New Zealand socio-economic index 2013. Wellington: Statistics New Zealand.* | |  |
| Income attribution | | incomeattributiont09 | | Continuous (1 items, range: 1 to 7) | | 1. If incomes were more equal, people would be less motivated to work hard. | |  |
| Relationship status | | relstatusl2 | | Categorical (Single, dating/engaged, partner, married, divorced/separated, widow, other ) | | 1. What is your relationship status? (e.g., single, married, de-facto, civil union, widowed, living together, etc.) | |  |
| Number of children | | childrennum | | Continuous (1 items, range: 0 to 26) | | 1. How many children have you given birth to, fathered, or adopted? | |  |
| Openness | | openness | | Continuous (4 items, range: 1 to 7) | | 1. Mini-IPIP6 Openness to Experience | |  |
| Neuroticism | | neuroticism | | Continuous (4 items, range: 1 to 7) | | 1. Mini-IPIP6 Neuroticism/Emotional Stability | |  |
| Extraversion | | extraversion | | Continuous (4 items, range: 1 to 7) | | 1. Mini-IPIP6 Extraversion | |  |
| Agreeableness | | agreeableness | | Continuous (4 items, range: 1 to 7) | | 1. Mini-IPIP6 Agreableness | |  |
| Conscientiousness | | conscientiousness | | Continuous (4 items, range: 1 to 7) | | 1. Mini-IPIP6 Conscientiousness | |  |
| Health locus of control | | hlthlocus | | Continuous (3 items, range: 1 to 7) | | *Wallston, K. A., Wallston, B. S., DeVellis, R. (1978). Development of the multidimensional health locus of control (MHLC*) scales   1. If I get sick, it is my own behavior which determines how soon I get well again. 2. If I take care of myself, I can avoid illness. 3. I am in control of my health. | |  |
| Political orientation | | polorient | | Continuous (1 items, range: 1 to 7) | | 1. Please rate how politically liberal versus conservative you see yourself as being. | |  |
| Hours/week putting on cosmetics | | hourscosmeticst09 | | Continuous (1 item, range: 0 to 100) | | 1. Hours… putting on make up/cosmetics last week | |  |
| Hours/week commuting | | hourscommutet09 | | Continuous (1 item, range: 0 to 100) | | 1. Hours… commuting/travelling last week | |  |
| Hours/week taking care of children | | hourschildrent09 | | Continuous (1 item, range: 0 to 168) | | 1. Hours… looking after children last week | |  |
| Hours/week doing housework | | hourshouseworkt09 | | Continuous (1 item, range: 0 to 168) | | 1. Hours… homework/cooking last week | |  |
| Hours/week working | | hoursworkt09 | | Continuous (1 item, range: 0 to 110) | | 1. Hours… working in paid employment last week | |  |

^1^ For all continuous outcomes, means were calculated with the scores of all selected items.

^2^ For these variables, lifetime smoking or being a cyberbullying victim was used as the prebaseline variable (Wave 1), and recent smoking or cyberbullying victim was used as the outcome (Wave 3).

**Table S2. Means and standard deviations of all continuous outcomes in 2019**

|  | **Total sample** | **Younger sample (age<40)** | **Older sample (age≥40)** |
| --- | --- | --- | --- |
|  | **n=11085** | **n=2296** | **n=8789** |
| **Psychological wellbeing** |  |  |  |
| Life satisfaction, *M* (*SD*) | 5.4 (1.2) | 5.2 (1.3) | 5.4 (1.2) |
| Self-steem, *M* (*SD*) | 5.2 (1.2) | 4.9 (1.4) | 5.3 (1.2) |
| Body satisfaction, *M* (*SD*) | 4.3 (1.7) | 4.0 (1.7) | 4.4 (1.6) |
| **Social wellbeing** |  |  |  |
| Sense of community, *M* (*SD*) | 4.4 (1.6) | 3.9 (1.6) | 4.5 (1.5) |
| Sense of belonging, *M* (*SD*) | 5.2 (1.1) | 5.0 (1.1) | 5.3 (1.0) |
| Social support, *M* (*SD*) | 6.0 (1.1) | 6.0 (1.1) | 6.0 (1.1) |
| **Character strengths and prosocial behavior** |  |  |  |
| Volunteering (hours/week), *M* (*SD*) | 1.6 (4.8) | 1.1 (3.6) | 1.7 (5.0) |
| Charity donations ($NZ), *M* (*SD*) | 1186.9 (8814.2) | 706.6 (3241.3) | 1311.6 (9750.6) |
| Self-control | 4.4 (1.4) | 3.8 (1.4) | 4.6 (1.4) |
| **Physical health** |  |  |  |
| Subjective health, *M* (*SD*) | 5.0 (1.1) | 5.0 (1.1) | 5.0 (1.1) |
| Sleep (hours/day), *M* (*SD*) | 6.9 (1.1) | 6.9 (1.1) | 6.9 (1.1) |
| Body mass index (kg/m^2^), *M* (*SD*) | 27.5 (5.8) | 26.7 (6.1) | 27.7 (5.7) |
| **Mental health** |  |  |  |
| Psychological distress, *M* (*SD*) | 0.8 (0.6) | 1.1 (0.7) | 0.7 (0.6) |
| **Health behavior** |  |  |  |
| Alcohol use, *M* (*SD*) | 1.3 (0.8) | 1.2 (0.8) | 1.3 (0.8) |
| Exercise (hours/week), *M* (*SD*) | 6.1 (7.4) | 4.8 (6.4) | 6.5 (7.6) |

**Table S3. Continuous *p*-values and robustness to unmeasured confounding (*E*-Values)^a^ for the associations between time spent in screen-based activities (2017) and subsequent outcomes (2019) among total sample**

| **Outcomes by theme** | **Total screen-based leisure time** | | **Browsing the internet** | | **Watching videos** | | **Watching or reading news** | | **Using social media** | | **Playing video games** | |
| --- | --- | --- | --- | --- | --- | --- | --- | --- | --- | --- | --- | --- |
|  | ***p*-value** | ***E*-v (l.95%CI)**^b^ | ***p*-value** | ***E*-v (l.95%CI)**^b^ | ***p*-value** | ***E*-v (l.95%CI)**^b^ | ***p*-value** | ***E*-v (l.95%CI)**^b^ | ***p*-value** | ***E*-v (l.95%CI)**^b^ | ***p*-value** | ***E*-v (l.95%CI)**^b^ |
| **Psychological wellbeing** |  |  |  |  |  |  |  |  |  |  |  |  |
| Life satisfaction | 0.088 | 1.13 (1.00) | 0.775 | 1.05 (1.00) | 0.027 | 1.15 (1.05) | 0.088 | 1.12 (1.00) | 0.865 | 1.04 (1.00) | 0.173 | 1.11 (1.00) |
| Self-esteem | 0.764 | 1.05 (1.00) | 0.829 | 1.04 (1.00) | 0.639 | 1.06 (1.00) | 0.569 | 1.07 (1.00) | 0.193 | 1.11 (1.00) | 0.210 | 1.11 (1.00) |
| Body satisfaction | 0.001 | 1.20 (1.12) | 0.005 | 1.18 (1.09) | 0.278 | 1.10 (1.00) | 0.045 | 1.14 (1.02) | 0.043 | 1.15 (1.02) | 0.270 | 1.10 (1.00) |
| **Social wellbeing** |  |  |  |  |  |  |  |  |  |  |  |  |
| Sense of community | 0.224 | 1.12 (1.00) | 0.068 | 1.15 (1.00) | 0.564 | 1.08 (1.00) | 0.678 | 1.06 (1.00) | 0.704 | 1.06 (1.00) | 0.349 | 1.10 (1.00) |
| Sense of belonging | 0.900 | 1.03 (1.00) | 0.483 | 1.08 (1.00) | 0.345 | 1.09 (1.00) | 0.660 | 1.06 (1.00) | 0.817 | 1.05 (1.00) | 0.819 | 1.04 (1.00) |
| Social support | 0.588 | 1.07 (1.00) | 0.302 | 1.10 (1.00) | 0.723 | 1.06 (1.00) | 0.071 | 1.13 (1.00) | 0.517 | 1.08 (1.00) | 0.331 | 1.10 (1.00) |
| **Social factors** |  |  |  |  |  |  |  |  |  |  |  |  |
| Recently cyberbullied | 0.673 | 1.14 (1.00) | 0.622 | 1.15 (1.00) | 0.826 | 1.10 (1.00) | 0.363 | 1.23 (1.00) | 0.848 | 1.09 (1.00) | 0.077 | 1.32 (1.00) |
| Engaged/married/entered a civil union^c^ | 0.798 | 1.13 (1.00) | 0.822 | 1.12 (1.00) | 0.113 | 1.45 (1.00) | 0.903 | 1.10 (1.00) | 0.965 | 1.05 (1.00) | 0.319 | 1.26 (1.00) |
| Divorced/separated^d^ | 0.118 | 1.49 (1.00) | 0.673 | 1.19 (1.00) | 0.368 | 1.34 (1.00) | 0.093 | 1.58 (1.00) | 0.074 | 1.63 (1.00) | 0.349 | 1.38 (1.00) |
| Assaulted/harassed/attacked | 0.587 | 1.15 (1.00) | 0.724 | 1.12 (1.00) | 0.489 | 1.18 (1.00) | 0.661 | 1.13 (1.00) | 0.625 | 1.13 (1.00) | 0.491 | 1.17 (1.00) |
| **Character strengths and prosocial behavior** |  |  |  |  |  |  |  |  |  |  |  |  |
| Volunteering (hours/week) | 0.026 | 1.18 (1.06) | 0.003 | 1.21 (1.11) | 0.847 | 1.05 (1.00) | 0.398 | 1.10 (1.00) | 0.146 | 1.14 (1.00) | 0.640 | 1.07 (1.00) |
| Charity donations, $NZ | 0.430 | 1.10 (1.00) | 0.686 | 1.07 (1.00) | 0.419 | 1.10 (1.00) | 0.294 | 1.11 (1.00) | 0.717 | 1.06 (1.00) | 0.646 | 1.07 (1.00) |
| Self-control | 0.003 | 1.18 (1.10) | 0.329 | 1.10 (1.00) | <.001 | 1.20 (1.13) | 0.219 | 1.10 (1.00) | 0.094 | 1.13 (1.00) | 0.166 | 1.11 (1.00) |
| **Physical health** |  |  |  |  |  |  |  |  |  |  |  |  |
| Subjective health | 0.004 | 1.18 (1.10) | 0.075 | 1.13 (1.00) | 0.019 | 1.16 (1.06) | 0.146 | 1.11 (1.00) | 0.862 | 1.04 (1.00) | 0.002 | 1.18 (1.10) |
| Sleep (hours/day) | 0.002 | 1.21 (1.12) | 0.019 | 1.17 (1.06) | 0.010 | 1.19 (1.08) | 0.972 | 1.02 (1.00) | 0.317 | 1.11 (1.00) | 0.014 | 1.17 (1.07) |
| Body mass index | <0.001 | 1.18 (1.13) | 0.105 | 1.10 (1.00) | <0.001 | 1.16 (1.11) | 0.055 | 1.10 (1.00) | 0.001 | 1.15 (1.09) | <0.001 | 1.16 (1.11) |
| Circulatory system diseases | 0.145 | 1.15 (1.00) | 0.039 | 1.19 (1.04) | 0.794 | 1.06 (1.00) | 0.862 | 1.04 (1.00) | 0.845 | 1.06 (1.00) | 0.271 | 1.13 (1.00) |
| Musculoskeletal system diseases | 0.515 | 1.16 (1.00) | 0.587 | 1.15 (1.00) | 0.080 | 1.26 (1.00) | 0.053 | 1.26 (1.00) | 0.316 | 1.22 (1.00) | 0.946 | 1.04 (1.00) |
| Consequences of external causes | 0.260 | 1.26 (1.00) | 0.552 | 1.18 (1.00) | 0.645 | 1.15 (1.00) | 0.159 | 1.24 (1.00) | 0.274 | 1.23 (1.00) | 0.766 | 1.12 (1.00) |
| **Mental health** |  |  |  |  |  |  |  |  |  |  |  |  |
| Psychological distress | 0.903 | 1.03 (1.00) | 0.935 | 1.03 (1.00) | 0.225 | 1.11 (1.00) | 0.398 | 1.09 (1.00) | 0.619 | 1.07 (1.00) | 0.514 | 1.08 (1.00) |
| Mental disorders | 0.339 | 1.13 (1.00) | 0.194 | 1.16 (1.00) | 0.928 | 1.04 (1.00) | 0.608 | 1.09 (1.00) | 0.685 | 1.07 (1.00) | 0.755 | 1.06 (1.00) |
| **Health behavior** |  |  |  |  |  |  |  |  |  |  |  |  |
| Alcohol use | 0.062 | 1.13 (1.00) | 0.253 | 1.10 (1.00) | 0.042 | 1.13 (1.02) | 0.866 | 1.03 (1.00) | 0.156 | 1.11 (1.00) | 0.888 | 1.03 (1.00) |
| Current smoking | 0.277 | 1.20 (1.00) | 0.909 | 1.06 (1.00) | 0.017 | 1.32 (1.12) | 0.659 | 1.12 (1.00) | 0.892 | 1.06 (1.00) | 0.875 | 1.07 (1.00) |
| Exercise (hours/week) | 0.001 | 1.22 (1.13) | 0.130 | 1.14 (1.00) | 0.014 | 1.19 (1.08) | 0.009 | 1.19 (1.09) | 0.024 | 1.18 (1.06) | 0.230 | 1.12 (1.00) |

^a^*E*-values for effect estimates are the minimum strength of association that an unmeasured confounder would need to have with both the exposure and the outcome variable to fully explain away the observed effect, after accounting for the measured covariates. The formula for calculating *E*-values can be found in VanderWeele and Ding (2017). ^b^*E*-values for the limit of the 95% CI closest to the null denote the minimum strength of association that an unmeasured confounder would need to have with both the exposure and the outcome variable to shift the confidence interval to include the null value, after accounting for the measured covariates. ^c^For this outcome, only participants married at pre-baseline were analyzed (n=6649). ^d^For this outcome, only participants that weren’t married at pre-baseline were analyzed (n=4436)

**Table S4. Continuous *p*-values and robustness to unmeasured confounding (*E*-Values)^a^ for the associations between time spent in screen-based activities (2017) and subsequent outcomes (2019) in subsample <40 years old**

| **Outcomes by theme** |  | **Total screen-based leisure time** | | **Browsing the internet** | | **Watching videos** | | **Watching or reading news** | | **Using social media** | | **Playing video games** | |
| --- | --- | --- | --- | --- | --- | --- | --- | --- | --- | --- | --- | --- | --- |
|  |  | ***p*-value** | ***E*-v (l.95%CI)**^b^ | ***p*-value** | ***E*-v (l.95%CI)**^b^ | ***p*-value** | ***E*-v (l.95%CI)**^b^ | ***p*-value** | ***E*-v (l.95%CI)**^b^ | ***p*-value** | ***E*-v (l.95%CI)**^b^ | ***p*-value** | ***E*-v (l.95%CI)**^b^ |
| **Psychological wellbeing** |  |  |  |  |  |  |  |  |  |  |  |  |  |
| Life satisfaction |  | 0.006 | 1.26 (1.12) | 0.227 | 1.15 (1.00) | 0.042 | 1.25 (1.04) | 0.084 | 1.24 (1.00) | 0.085 | 1.17 (1.00) | 0.001 | 1.28 (1.16) |
| Self-esteem |  | 0.746 | 1.08 (1.00) | 0.616 | 1.09 (1.00) | 0.798 | 1.07 (1.00) | 0.974 | 1.03 (1.00) | 0.392 | 1.12 (1.00) | 0.032 | 1.21 (1.06) |
| Body satisfaction |  | 0.523 | 1.11 (1.00) | 0.732 | 1.08 (1.00) | 0.729 | 1.09 (1.00) | 0.604 | 1.12 (1.00) | 0.812 | 1.06 (1.00) | 0.061 | 1.20 (1.00) |
| **Social wellbeing** |  |  |  |  |  |  |  |  |  |  |  |  |  |
| Sense of community |  | 0.264 | 1.16 (1.00) | 0.093 | 1.20 (1.00) | 0.408 | 1.16 (1.00) | 0.157 | 1.23 (1.00) | 0.981 | 1.02 (1.00) | 0.348 | 1.14 (1.00) |
| Sense of belonging |  | 0.037 | 1.21 (1.05) | 0.175 | 1.16 (1.00) | 0.014 | 1.27 (1.11) | 0.027 | 1.27 (1.08) | 0.864 | 1.05 (1.00) | 0.206 | 1.15 (1.00) |
| Social support |  | 0.957 | 1.03 (1.00) | 0.891 | 1.05 (1.00) | 0.664 | 1.10 (1.00) | 0.211 | 1.19 (1.00) | 0.406 | 1.11 (1.00) | 0.876 | 1.05 (1.00) |
| **Social factors** |  |  |  |  |  |  |  |  |  |  |  |  |  |
| Recently cyberbullied |  | 0.663 | 1.33 (1.00) | 0.477 | 1.28 (1.00) | 0.892 | 1.12 (1.00) | 0.471 | 1.42 (1.00) | 0.484 | 1.33 (1.00) | 0.102 | 1.49 (1.00) |
| Engaged/married/entered a civil union^c^ |  | 0.746 | 1.26 (1.00) | 0.941 | 1.07 (1.00) | 0.171 | 1.58 (1.00) | 0.867 | 1.14 (1.00) | 0.744 | 1.15 (1.00) | 0.722 | 1.18 (1.00) |
| Divorced/separated^d^ |  | 0.785 | 1.48 (1.00) | 0.683 | 1.37 (1.00) | 0.315 | 1.64 (1.00) | 0.897 | 1.20 (1.00) | 0.280 | 1.80 (1.00) | 0.724 | 1.40 (1.00) |
| Assaulted/harassed/attacked |  | 0.584 | 1.32 (1.00) | 0.988 | 1.03 (1.00) | 0.144 | 1.48 (1.00) | 0.997 | 1.02 (1.00) | 0.883 | 1.09 (1.00) | 0.899 | 1.09 (1.00) |
| **Character strengths and prosocial behavior** |  |  |  |  |  |  |  |  |  |  |  |  |  |
| Volunteering (hours/week) |  | 0.847 | 1.06 (1.00) | 0.595 | 1.09 (1.00) | 0.453 | 1.13 (1.00) | 0.310 | 1.17 (1.00) | 0.136 | 1.15 (1.00) | 0.193 | 1.15 (1.00) |
| Charity donations, $NZ |  | 0.680 | 1.06 (1.00) | 0.495 | 1.07 (1.00) | 0.734 | 1.06 (1.00) | 0.573 | 1.08 (1.00) | 0.633 | 1.06 (1.00) | 0.869 | 1.03 (1.00) |
| Self-control |  | 0.233 | 1.15 (1.00) | 0.947 | 1.03 (1.00) | 0.145 | 1.20 (1.00) | 0.213 | 1.19 (1.00) | 0.070 | 1.18 (1.00) | 0.869 | 1.05 (1.00) |
| **Physical health** |  |  |  |  |  |  |  |  |  |  |  |  |  |
| Subjective health |  | 0.135 | 1.17 (1.00) | 0.359 | 1.13 (1.00) | 0.259 | 1.17 (1.00) | 0.548 | 1.12 (1.00) | 0.860 | 1.05 (1.00) | 0.009 | 1.23 (1.10) |
| Sleep (hours/day) |  | 0.240 | 1.16 (1.00) | 0.376 | 1.14 (1.00) | 0.109 | 1.23 (1.00) | 0.100 | 1.24 (1.00) | 0.462 | 1.11 (1.00) | 0.671 | 1.09 (1.00) |
| Body mass index |  | <0.001 | 1.23 (1.14) | 0.016 | 1.17 (1.07) | 0.005 | 1.22 (1.11) | 0.431 | 1.11 (1.00) | 0.010 | 1.16 (1.07) | 0.027 | 1.16 (1.05) |
| Circulatory system diseases |  | 0.020 | 1.74 (1.21) | 0.114 | 1.56 (1.00) | 0.007 | 1.90 (1.34) | 0.581 | 1.35 (1.00) | 0.546 | 1.28 (1.00) | 0.403 | 1.39 (1.00) |
| Musculoskeletal system diseases |  | 0.329 | 1.84 (1.00) | 0.702 | 1.25 (1.00) | 0.990 | 1.04 (1.00) | 0.121 | 1.63 (1.00) | 0.118 | 1.70 (1.00) | 0.064 | 1.88 (1.00) |
| Consequences of external causes |  | 0.897 | 1.18 (1.00) | 0.965 | 1.07 (1.00) | 0.533 | 1.34 (1.00) | 0.738 | 1.30 (1.00) | 0.738 | 1.18 (1.00) | 0.354 | 1.52 (1.00) |
| **Mental health** |  |  |  |  |  |  |  |  |  |  |  |  |  |
| Psychological distress |  | 0.207 | 1.17 (1.00) | 0.647 | 1.09 (1.00) | 0.588 | 1.12 (1.00) | 0.123 | 1.23 (1.00) | 0.194 | 1.15 (1.00) | 0.304 | 1.14 (1.00) |
| Mental disorders |  | 0.308 | 1.26 (1.00) | 0.466 | 1.22 (1.00) | 0.573 | 1.20 (1.00) | 0.332 | 1.34 (1.00) | 0.409 | 1.21 (1.00) | 0.988 | 1.02 (1.00) |
| **Health behavior** |  |  |  |  |  |  |  |  |  |  |  |  |  |
| Alcohol use |  | 0.728 | 1.08 (1.00) | 0.126 | 1.17 (1.00) | 0.050 | 1.23 (1.01) | 0.075 | 1.23 (1.00) | 0.559 | 1.09 (1.00) | 0.903 | 1.04 (1.00) |
| Current smoking |  | 0.444 | 1.40 (1.00) | 0.954 | 1.06 (1.00) | 0.160 | 1.43 (1.00) | 0.205 | 1.46 (1.00) | 0.336 | 1.27 (1.00) | 0.229 | 1.37 (1.00) |
| Exercise (hours/week) |  | 0.041 | 1.23 (1.04) | 0.122 | 1.18 (1.00) | 0.679 | 1.10 (1.00) | 0.661 | 1.11 (1.00) | 0.061 | 1.19 (1.00) | 0.007 | 1.25 (1.12) |

^a^*E*-values for effect estimates are the minimum strength of association that an unmeasured confounder would need to have with both the exposure and the outcome variable to fully explain away the observed effect, after accounting for the measured covariates. The formula for calculating *E*-values can be found in VanderWeele and Ding (2017). ^b^*E*-values for the limit of the 95% CI closest to the null denote the minimum strength of association that an unmeasured confounder would need to have with both the exposure and the outcome variable to shift the confidence interval to include the null value, after accounting for the measured covariates. ^c^For this outcome, only participants married at pre-baseline were analyzed (n=968). ^d^For this outcome, only participants that weren’t married at pre-baseline were analyzed (n=1328)

**Table S5. Continuous *p*-values and robustness to unmeasured confounding (*E*-Values)^a^ for the associations time in screen-based activities in 2017 and subsequent outcomes in 2019 among subsample** **≥40 years old**

| **Outcomes by theme** | **Total screen-based leisure time** | | **Browsing the internet** | | **Watching videos** | | **Watching or reading news** | | **Using social media** | | **Playing video games** | |
| --- | --- | --- | --- | --- | --- | --- | --- | --- | --- | --- | --- | --- |
|  | ***p*-value** | ***E*-v (l.95%CI)**^b^ | ***p*-value** | ***E*-v (l.95%CI)**^b^ | ***p*-value** | ***E*-v (l.95%CI)**^b^ | ***p*-value** | ***E*-v (l.95%CI)**^b^ | ***p*-value** | ***E*-v (l.95%CI)**^b^ | ***p*-value** | ***E*-v (l.95%CI)**^b^ |
| **Psychological wellbeing** |  |  |  |  |  |  |  |  |  |  |  |  |
| Life satisfaction | 0.738 | 1.06 (1.00) | 0.722 | 1.06 (1.00) | 0.115 | 1.13 (1.00) | 0.282 | 1.10 (1.00) | 0.302 | 1.12 (1.00) | 0.315 | 1.10 (1.00) |
| Self-esteem | 0.428 | 1.09 (1.00) | 0.811 | 1.05 (1.00) | 0.529 | 1.08 (1.00) | 0.441 | 1.08 (1.00) | 0.616 | 1.08 (1.00) | 0.645 | 1.07 (1.00) |
| Body satisfaction | 0.001 | 1.23 (1.14) | 0.001 | 1.22 (1.13) | 0.373 | 1.10 (1.00) | 0.030 | 1.15 (1.04) | 0.002 | 1.23 (1.13) | 0.715 | 1.06 (1.00) |
| **Social wellbeing** |  |  |  |  |  |  |  |  |  |  |  |  |
| Sense of community | 0.522 | 1.09 (1.00) | 0.325 | 1.11 (1.00) | 0.790 | 1.05 (1.00) | 0.794 | 1.05 (1.00) | 0.620 | 1.08 (1.00) | 0.428 | 1.10 (1.00) |
| Sense of belonging | 0.254 | 1.12 (1.00) | 0.861 | 1.04 (1.00) | 0.033 | 1.16 (1.04) | 0.868 | 1.04 (1.00) | 0.647 | 1.08 (1.00) | 0.336 | 1.10 (1.00) |
| Social support | 0.373 | 1.11 (1.00) | 0.129 | 1.14 (1.00) | 0.529 | 1.08 (1.00) | 0.115 | 1.13 (1.00) | 0.905 | 1.04 (1.00) | 0.370 | 1.10 (1.00) |
| **Social factors** |  |  |  |  |  |  |  |  |  |  |  |  |
| Recently cyberbullied | 0.285 | 1.44 (1.00) | 0.342 | 1.25 (1.00) | 0.726 | 1.15 (1.00) | 0.682 | 1.15 (1.00) | 0.147 | 1.34 (1.00) | 0.396 | 1.25 (1.00) |
| Engaged/married/entered a civil union^c^ | 0.909 | 1.19 (1.00) | 0.619 | 1.30 (1.00) | 0.404 | 1.42 (1.00) | 0.854 | 1.18 (1.00) | 0.855 | 1.18 (1.00) | 0.431 | 1.27 (1.00) |
| Divorced/separated^d^ | 0.113 | 1.99 (1.00) | 0.898 | 1.11 (1.00) | 0.123 | 1.55 (1.00) | 0.121 | 1.61 (1.00) | 0.076 | 1.81 (1.00) | 0.428 | 1.38 (1.00) |
| Assaulted/harassed/attacked | 0.725 | 1.21 (1.00) | 0.915 | 1.07 (1.00) | 0.491 | 1.20 (1.00) | 0.765 | 1.12 (1.00) | 0.278 | 1.25 (1.00) | 0.609 | 1.17 (1.00) |
| **Character strengths and prosocial behavior** |  |  |  |  |  |  |  |  |  |  |  |  |
| Volunteering (hours/week) | 0.006 | 1.23 (1.11) | 0.001 | 1.26 (1.16) | 0.660 | 1.08 (1.00) | 0.187 | 1.13 (1.00) | 0.351 | 1.14 (1.00) | 0.963 | 1.03 (1.00) |
| Charity donations, $NZ | 0.258 | 1.14 (1.00) | 0.404 | 1.12 (1.00) | 0.363 | 1.12 (1.00) | 0.485 | 1.10 (1.00) | 0.827 | 1.06 (1.00) | 0.499 | 1.11 (1.00) |
| Self-control | 0.004 | 1.20 (1.10) | 0.209 | 1.12 (1.00) | 0.001 | 1.21 (1.13) | 0.373 | 1.09 (1.00) | 0.386 | 1.11 (1.00) | 0.222 | 1.12 (1.00) |
| **Physical health** |  |  |  |  |  |  |  |  |  |  |  |  |
| Subjective health | 0.021 | 1.18 (1.06) | 0.128 | 1.14 (1.00) | 0.078 | 1.14 (1.00) | 0.299 | 1.10 (1.00) | 0.880 | 1.04 (1.00) | 0.033 | 1.17 (1.04) |
| Sleep (hours/day) | 0.006 | 1.21 (1.10) | 0.024 | 1.19 (1.06) | 0.095 | 1.15 (1.00) | 0.966 | 1.02 (1.00) | 0.616 | 1.09 (1.00) | 0.004 | 1.21 (1.11) |
| Body mass index | 0.004 | 1.15 (1.08) | 0.994 | 1.01 (1.00) | 0.006 | 1.14 (1.07) | 0.075 | 1.10 (1.00) | 0.015 | 1.15 (1.06) | <0.001 | 1.18 (1.12) |
| Circulatory system diseases | 0.328 | 1.19 (1.00) | 0.110 | 1.26 (1.00) | 0.801 | 1.08 (1.00) | 0.990 | 1.02 (1.00) | 0.976 | 1.03 (1.00) | 0.256 | 1.20 (1.00) |
| Musculoskeletal system diseases | 0.149 | 1.41 (1.00) | 0.950 | 1.05 (1.00) | 0.042 | 1.30 (1.05) | 0.064 | 1.26 (1.00) | 0.790 | 1.11 (1.00) | 0.431 | 1.17 (1.00) |
| Consequences of external causes | 0.230 | 1.47 (1.00) | 0.441 | 1.23 (1.00) | 0.760 | 1.13 (1.00) | 0.102 | 1.27 (1.00) | 0.410 | 1.23 (1.00) | 0.998 | 1.01 (1.00) |
| **Mental health** |  |  |  |  |  |  |  |  |  |  |  |  |
| Psychological distress | 0.173 | 1.13 (1.00) | 0.564 | 1.08 (1.00) | 0.046 | 1.15 (1.02) | 0.905 | 1.03 (1.00) | 0.540 | 1.09 (1.00) | 0.973 | 1.02 (1.00) |
| Mental disorders | 0.827 | 1.10 (1.00) | 0.369 | 1.22 (1.00) | 0.692 | 1.13 (1.00) | 0.859 | 1.07 (1.00) | 0.621 | 1.14 (1.00) | 0.794 | 1.10 (1.00) |
| **Health behavior** |  |  |  |  |  |  |  |  |  |  |  |  |
| Alcohol use | 0.005 | 1.18 (1.09) | 0.018 | 1.16 (1.06) | 0.133 | 1.12 (1.00) | 0.173 | 1.11 (1.00) | 0.023 | 1.17 (1.06) | 0.596 | 1.07 (1.00) |
| Current smoking | 0.586 | 1.23 (1.00) | 0.983 | 1.03 (1.00) | 0.091 | 1.28 (1.00) | 0.989 | 1.02 (1.00) | 0.440 | 1.20 (1.00) | 0.929 | 1.05 (1.00) |
| Exercise (hours/week) | 0.006 | 1.23 (1.11) | 0.425 | 1.11 (1.00) | 0.006 | 1.22 (1.11) | 0.002 | 1.22 (1.12) | 0.167 | 1.16 (1.00) | 0.761 | 1.07 (1.00) |

^a^*E*-values for effect estimates are the minimum strength of association that an unmeasured confounder would need to have with both the exposure and the outcome variable to fully explain away the observed effect, after accounting for the measured covariates. The formula for calculating *E*-values can be found in VanderWeele and Ding (2017). ^b^*E*-values for the limit of the 95% CI closest to the null denote the minimum strength of association that an unmeasured confounder would need to have with both the exposure and the outcome variable to shift the confidence interval to include the null value, after accounting for the measured covariates. ^c^For this outcome, only participants married at pre-baseline were analyzed (n=5681). ^d^For this outcome, only participants that weren’t married at pre-baseline were analyzed (n=3108

**Table S6. Quartiles of total screen-based leisure time (2017) and subsequent outcomes (2019) in total sample**

|  | **Groups of screen-based leisure time** | | | | | | | | | |
| --- | --- | --- | --- | --- | --- | --- | --- | --- | --- | --- |
|  | **Quartile 1**  (<18 hours) | **Quartile 2**  (18 to 28 hours) | |  | **Quartile 3**  (29 to 42 hours) | |  | **Quartile 4**  (>43 hours) | |  |
| **Outcomes by theme** | **β/OR (95%CI)^a,b,c^** | **β/OR (95%CI)^a,b,c^** | ***p*-value** | ***E*-value**  **(lower 95%CI)** | **β/OR (95%CI)^a,b,c^** | ***p*-value** | ***E*-value**  **(lower 95%CI)** | **β/OR (95%CI)^a,b,c^** | ***p*-value** | ***E*-value**  **(lower 95%CI)** |
| **Psychological wellbeing** |  |  |  |  |  |  |  |  |  |  |
| Life satisfaction | 0 (ref) | -0.04 (-0.08, -0.00)* | 0.038 | 1.23 (1.05) | -0.01 (-0.05, 0.03) | 0.758 | 1.08 (1.00) | -0.03 (-0.08, 0.01) | 0.126 | 1.21 (1.00) |
| Self-esteem | 0 (ref) | 0.03 (-0.01, 0.06) | 0.156 | 1.18 (1.00) | 0.02 (-0.02, 0.05) | 0.399 | 1.14 (1.00) | 0.03 (-0.01, 0.07) | 0.144 | 1.20 (1.00) |
| Body satisfaction | 0 (ref) | -0.01 (-0.05, 0.02) | 0.462 | 1.13 (1.00) | -0.05 (-0.10, -0.01)* | 0.012 | 1.28 (1.12) | -0.09 (-0.14, -0.04)** | <0.001 | 1.39 (1.25) |
| **Social wellbeing** |  |  |  |  |  |  |  |  |  |  |
| Sense of community | 0 (ref) | -0.02 (-0.06, 0.02) | 0.396 | 1.15 (1.00) | -0.03 (-0.07, 0.02) | 0.266 | 1.18 (1.00) | -0.06 (-0.11, -0.01)* | 0.021 | 1.30 (1.10) |
| Sense of belonging | 0 (ref) | -0.00 (-0.04, 0.04) | 0.981 | 1.02 (1.00) | -0.03 (-0.07, 0.01) | 0.117 | 1.21 (1.00) | 0.01 (-0.04, 0.05) | 0.704 | 1.10 (1.00) |
| Social support | 0 (ref) | -0.00 (-0.04, 0.04) | 0.857 | 1.06 (1.00) | -0.01 (-0.06, 0.03) | 0.504 | 1.13 (1.00) | 0.01 (-0.04, 0.05) | 0.814 | 1.08 (1.00) |
| **Social factors** |  |  |  |  |  |  |  |  |  |  |
| Recently cyberbullied | 1 (ref) | 0.99 (0.65, 1.50) | 0.945 | 1.09 (1.00) | 1.42 (0.94, 2.15) | 0.092 | 1.67 (1.00) | 1.33 (0.86, 2.06) | 0.206 | 1.57 (1.00) |
| Engaged/married/entered a civil union^d^ | 1 (ref) | 0.86 (0.49, 1.52) | 0.610 | 1.36 (1.00) | 0.83 (0.46, 1.49) | 0.525 | 1.43 (1.00) | 1.05 (0.58, 1.90) | 0.882 | 1.18 (1.00) |
| Divorced/separated^e^ | 1 (ref) | 1.51 (0.87, 2.60) | 0.141 | 1.75 (1.00) | 1.41 (0.77, 2.57) | 0.261 | 1.66 (1.00) | 0.79 (0.39, 1.60) | 0.505 | 1.51 (1.00) |
| Assaulted/harassed/attacked | 1 (ref) | 1.17 (0.85, 1.60) | 0.340 | 1.37 (1.00) | 1.19 (0.86, 1.67) | 0.298 | 1.41 (1.00) | 1.05 (0.73, 1.51) | 0.804 | 1.18 (1.00) |
| **Character strengths and prosocial behavior** |  |  |  |  |  |  |  |  |  |  |
| Volunteering (hours/week) | 0 (ref) | 0.02 (-0.03, 0.07) | 0.431 | 1.15 (1.00) | 0.06 (0.01, 0.11)* | 0.025 | 1.30 (1.09) | 0.09 (0.03, 0.14)* | 0.003 | 1.38 (1.19) |
| Charity donations, amount | 0 (ref) | -0.01 (-0.07, 0.04) | 0.621 | 1.12 (1.00) | -0.02 (-0.08, 0.03) | 0.357 | 1.18 (1.00) | -0.03 (-0.09, 0.03) | 0.383 | 1.19 (1.00) |
| Self-control | 0 (ref) | -0.03 (-0.07, 0.01) | 0.090 | 1.21 (1.00) | -0.07 (-0.11, -0.03)** | 0.001 | 1.32 (1.18) | -0.08 (-0.13, -0.04)** | <0.001 | 1.37 (1.23) |
| **Physical health** |  |  |  |  |  |  |  |  |  |  |
| Subjective health | 0 (ref) | -0.02 (-0.06, 0.02) | 0.313 | 1.15 (1.00) | -0.05 (-0.10, -0.01)* | 0.008 | 1.28 (1.13) | -0.07 (-0.12, -0.03)** | 0.001 | 1.34 (1.19) |
| Sleep (hours/day) | 0 (ref) | -0.00 (-0.05, 0.04) | 0.836 | 1.07 (1.00) | -0.05 (-0.09, 0.00) | 0.058 | 1.25 (1.00) | -0.05 (-0.10, 0.01) | 0.077 | 1.26 (1.00) |
| Body mass index (kg/m^2^) | 0 (ref) | -0.00 (-0.03, 0.02) | 0.795 | 1.06 (1.00) | 0.02 (-0.01, 0.04) | 0.245 | 1.13 (1.00) | 0.05 (0.03, 0.08)** | <0.001 | 1.28 (1.18) |
| Circulatory system diseases | 1 (ref) | 1.01 (0.90, 1.13) | 0.860 | 1.08 (1.00) | 1.04 (0.93, 1.17) | 0.489 | 1.17 (1.00) | 1.06 (0.94, 1.20) | 0.344 | 1.21 (1.00) |
| Musculoskeletal system diseases | 1 (ref) | 1.00 (0.77, 1.29) | 0.978 | 1.04 (1.00) | 1.07 (0.82, 1.41) | 0.601 | 1.23 (1.00) | 1.18 (0.88, 1.57) | 0.265 | 1.39 (1.00) |
| Consequences of external causes | 1 (ref) | 1.35 (0.93, 1.97) | 0.117 | 1.60 (1.00) | 1.34 (0.89, 2.00) | 0.159 | 1.58 (1.00) | 1.29 (0.83, 2.00) | 0.266 | 1.52 (1.00) |
| **Mental health** |  |  |  |  |  |  |  |  |  |  |
| Psychological distress | 0 (ref) | -0.02 (-0.05, 0.02) | 0.400 | 1.14 (1.00) | -0.03 (-0.07, 0.01) | 0.110 | 1.21 (1.00) | -0.03 (-0.07, 0.01) | 0.190 | 1.20 (1.00) |
| Mental disorders | 1 (ref) | 0.94 (0.82, 1.08) | 0.413 | 1.20 (1.00) | 0.94 (0.81, 1.08) | 0.377 | 1.22 (1.00) | 0.95 (0.82, 1.11) | 0.522 | 1.19 (1.00) |
| **Health behavior** |  |  |  |  |  |  |  |  |  |  |
| Alcohol use | 0 (ref) | 0.01 (-0.02, 0.05) | 0.401 | 1.13 (1.00) | 0.01 (-0.03, 0.05) | 0.559 | 1.11 (1.00) | 0.04 (0.00, 0.08)* | 0.029 | 1.25 (1.07) |
| Current smoking | 1 (ref) | 0.85 (0.64, 1.13) | 0.252 | 1.39 (1.00) | 0.82 (0.61, 1.11) | 0.203 | 1.44 (1.00) | 0.99 (0.73, 1.34) | 0.945 | 1.08 (1.00) |
| Exercise (hours/week) | 0 (ref) | -0.01 (-0.06, 0.04) | 0.684 | 1.11 (1.00) | 0.04 (-0.01, 0.09) | 0.150 | 1.23 (1.00) | 0.04 (-0.02, 0.09) | 0.225 | 1.22 (1.00) |

Abbreviations: OR, odds ratio; CI, confidence interval.

* *p* < 0.05 before Bonferroni correction.

** *p* < 0.05 after Bonferroni correction (the *p*-value cutoff for Bonferroni correction is *p* = 0.05/24 outcomes = p < 0.002).

^a^ All continuous outcomes (life satisfaction, self-esteem, sense of community, sense of belonging, social support, volunteering hours, amount of charity donations, self-control, subjective health, psychological distress, hours of sleep, body mass index, body satisfaction, alcohol intake, hours of exercise) were standardized (mean = 0, standard deviation = 1), and the standardized β was calculated through a lineal regression. For binary outcomes with a prevalence <10%, logistical regression models were run to calculate Odds Ratios (been cyberbullied, engaged, married or entered a civil union, divorced or separated, current smoking, diagnose of musculoskeletal system diseases, and consequences of external causes outcomes). For binary outcomes with a prevalence >10% (diagnose of mental health or circulatory system diseases), generalized linear models with a log link and Poisson distribution were run to calculate Relative Risks

^b^ All models controlled for pre-baseline covariates (age, gender, ethnicity, sexual orientation, been born in New Zealand, resident in urban area, New Zealand socioeconomic index, occupation, income attribution, educational level, religion, relationship status, political orientation, openness, neuroticism, extraversion, conscientiousness, agreeableness, health locus of control, number of children, hours putting on cosmetics hours commuting, hours looking after children, hours of housework, hours working, pre-baseline screen-based time activities and pre-baseline levels for all outcomes).

^c^ Regression was performed by using the multiple imputed sample (n=11085).

^d^ For this outcome, only participants married at pre-baseline were analyzed (n=6649)

^e^ For this outcome, only participants that weren’t married at pre-baseline were analyzed (n=4436)

**Table S7. Quartiles of total screen-based leisure time (2017) and subsequent outcomes (2019) in subsample < 40 years old**

|  | **Groups of screen-based leisure time** | | | | | | | | | |
| --- | --- | --- | --- | --- | --- | --- | --- | --- | --- | --- |
|  | **Quartile 1**  (<18 hours) | **Quartile 2**  (18 to 28 hours) | | | **Quartile 3**  (29 to 42 hours) | | | **Quartile 4**  (>43 hours) | | |
| **Outcomes by theme** | **β/OR (95%CI)^a,b,c^** | **β/OR (95%CI)^a,b,c^** | ***p*-value** | ***E*-value**  **(lower 95%CI)** | **β/OR (95%CI)^a,b,c^** | ***p*-value** | ***E*-value**  **(lower 95%CI)** | **β/OR (95%CI)^a,b,c^** | ***p*-value** | ***E*-value**  **(lower 95%CI)** |
| **Psychological wellbeing** |  |  |  |  |  |  |  |  |  |  |
| Life satisfaction | 0 (ref) | -0.01 (-0.11, 0.09) | 0.871 | 1.09 (1.00) | -0.01 (-0.11, 0.09) | 0.842 | 1.11 (1.00) | -0.08 (-0.19, 0.03) | 0.137 | 1.36 (1.00) |
| Self-esteem | 0 (ref) | 0.04 (-0.06, 0.13) | 0.453 | 1.22 (1.00) | 0.04 (-0.06, 0.14) | 0.388 | 1.25 (1.00) | -0.01 (-0.12, 0.09) | 0.824 | 1.12 (1.00) |
| Body satisfaction | 0 (ref) | -0.03 (-0.13, 0.07) | 0.588 | 1.18 (1.00) | -0.06 (-0.16, 0.05) | 0.289 | 1.28 (1.00) | -0.13 (-0.24, -0.03)* | 0.015 | 1.50 (1.18) |
| **Social wellbeing** |  |  |  |  |  |  |  |  |  |  |
| Sense of community | 0 (ref) | -0.05 (-0.16, 0.06) | 0.354 | 1.28 (1.00) | -0.07 (-0.19, 0.04) | 0.225 | 1.34 (1.00) | -0.12 (-0.24, 0.00) | 0.058 | 1.47 (1.00) |
| Sense of belonging | 0 (ref) | -0.02 (-0.12, 0.07) | 0.635 | 1.17 (1.00) | -0.10 (-0.20, -0.00)* | 0.049 | 1.42 (1.03) | -0.07 (-0.17, 0.03) | 0.169 | 1.34 (1.00) |
| Social support | 0 (ref) | -0.02 (-0.12, 0.08) | 0.729 | 1.14 (1.00) | -0.03 (-0.14, 0.07) | 0.503 | 1.22 (1.00) | 0.02 (-0.09, 0.12) | 0.763 | 1.14 (1.00) |
| **Social factors** |  |  |  |  |  |  |  |  |  |  |
| Recently cyberbullied | 1 (ref) | 1.31 (0.46, 3.72) | 0.617 | 1.55 (1.00) | 1.24 (0.42, 3.60) | 0.696 | 1.47 (1.00) | 0.84 (0.28, 2.54) | 0.762 | 1.40 (1.00) |
| Engaged/married/entered a civil union^d^ | 1 (ref) | 0.99 (0.44, 2.19) | 0.973 | 1.09 (1.00) | 0.83 (0.37, 1.87) | 0.652 | 1.43 (1.00) | 1.04 (0.46, 2.32) | 0.933 | 1.15 (1.00) |
| Divorced/separated^e^ | 1 (ref) | 4.64 (0.67, 31.96) | 0.119 | 3.73 (1.00) | 8.39 (1.28, 54.79)* | 0.026 | 5.24 (1.52) | 2.42 (0.26, 22.35) | 0.435 | 2.49 (1.00) |
| Assaulted/harassed/attacked | 1 (ref) | 0.93 (0.48, 1.81) | 0.826 | 1.24 (1.00) | 1.16 (0.58, 2.32) | 0.673 | 1.37 (1.00) | 0.65 (0.31, 1.34) | 0.239 | 1.80 (1.00) |
| **Character strengths and prosocial behavior** |  |  |  |  |  |  |  |  |  |  |
| Volunteering (hours/week) | 0 (ref) | -0.01 (-0.10, 0.08) | 0.792 | 1.12 (1.00) | 0.02 (-0.08, 0.11) | 0.710 | 1.15 (1.00) | 0.01 (-0.09, 0.11) | 0.792 | 1.12 (1.00) |
| Charity donations, amount | 0 (ref) | 0.01 (-0.03, 0.05) | 0.649 | 1.11 (1.00) | 0.02 (-0.02, 0.07) | 0.362 | 1.16 (1.00) | 0.01 (-0.03, 0.06) | 0.586 | 1.12 (1.00) |
| Self-control | 0 (ref) | -0.08 (-0.18, 0.01) | 0.085 | 1.37 (1.00) | -0.11 (-0.21, -0.01)* | 0.030 | 1.44 (1.11) | -0.09 (-0.19, 0.02) | 0.095 | 1.38 (1.00) |
| **Physical health** |  |  |  |  |  |  |  |  |  |  |
| Subjective health | 0 (ref) | -0.02 (-0.11, 0.07) | 0.680 | 1.15 (1.00) | -0.10 (-0.19, 0.00) | 0.052 | 1.40 (1.00) | -0.13 (-0.23, -0.03)* | 0.013 | 1.49 (1.18) |
| Sleep (hours/day) | 0 (ref) | 0.07 (-0.03, 0.18) | 0.181 | 1.34 (1.00) | 0.01 (-0.11, 0.12) | 0.921 | 1.08 (1.00) | 0.06 (-0.06, 0.17) | 0.332 | 1.29 (1.00) |
| Body mass index (kg/m^2^) | 0 (ref) | 0.02 (-0.04, 0.08) | 0.491 | 1.16 (1.00) | 0.01 (-0.05, 0.08) | 0.642 | 1.13 (1.00) | 0.06 (-0.00, 0.12) | 0.067 | 1.30 (1.00) |
| Circulatory system diseases | 1 (ref) | 1.33 (0.71, 2.50) | 0.371 | 1.58 (1.00) | 1.04 (0.55, 1.99) | 0.897 | 1.17 (1.00) | 1.51 (0.79, 2.88) | 0.216 | 1.76 (1.00) |
| Musculoskeletal system diseases | 1 (ref) | 0.87 (0.24, 3.11) | 0.831 | 1.35 (1.00) | 1.06 (0.29, 3.82) | 0.934 | 1.20 (1.00) | 0.69 (0.18, 2.63) | 0.591 | 1.69 (1.00) |
| Consequences of external causes | 1 (ref) | 1.13 (0.40, 3.19) | 0.819 | 1.32 (1.00) | 0.54 (0.16, 1.85) | 0.324 | 2.07 (1.00) | 0.61 (0.18, 2.13) | 0.440 | 1.87 (1.00) |
| **Mental health** |  |  |  |  |  |  |  |  |  |  |
| Psychological distress | 0 (ref) | -0.08 (-0.18, 0.02) | 0.109 | 1.37 (1.00) | -0.05 (-0.16, 0.06) | 0.344 | 1.27 (1.00) | -0.09 (-0.20, 0.02) | 0.104 | 1.39 (1.00) |
| Mental disorders | 1 (ref) | 1.03 (0.78, 1.35) | 0.851 | 1.13 (1.00) | 1.00 (0.76, 1.32) | 0.993 | 1.03 (1.00) | 1.00 (0.76, 1.32) | 0.983 | 1.04 (1.00) |
| **Health behavior** |  |  |  |  |  |  |  |  |  |  |
| Alcohol use | 0 (ref) | 0.01 (-0.08, 0.09) | 0.910 | 1.07 (1.00) | 0.00 (-0.09, 0.09) | 0.987 | 1.03 (1.00) | 0.03 (-0.07, 0.13) | 0.537 | 1.20 (1.00) |
| Current smoking | 1 (ref) | 0.77 (0.34, 1.74) | 0.535 | 1.53 (1.00) | 0.86 (0.37, 2.00) | 0.730 | 1.37 (1.00) | 1.08 (0.48, 2.41) | 0.860 | 1.23 (1.00) |
| Exercise (hours/week) | 0 (ref) | 0.02 (-0.09, 0.12) | 0.772 | 1.13 (1.00) | 0.01 (-0.10, 0.12) | 0.878 | 1.10 (1.00) | 0.02 (-0.10, 0.13) | 0.749 | 1.15 (1.00) |

Abbreviations: OR, odds ratio; CI, confidence interval.

* *p* < 0.05 before Bonferroni correction.

** *p* < 0.05 after Bonferroni correction (the *p*-value cutoff for Bonferroni correction is *p* = 0.05/24 outcomes = p < 0.002).

^a^ All continuous outcomes (life satisfaction, self-esteem, sense of community, sense of belonging, social support, volunteering hours, amount of charity donations, self-control, subjective health, psychological distress, hours of sleep, body mass index, body satisfaction, alcohol intake, hours of exercise) were standardized (mean = 0, standard deviation = 1), and the standardized β was calculated through a lineal regression. For binary outcomes with a prevalence <10%, logistical regression models were run to calculate Odds Ratios (been cyberbullied, engaged, married or entered a civil union, divorced or separated, current smoking, diagnose of musculoskeletal system diseases, and consequences of external causes outcomes). For binary outcomes with a prevalence >10% (diagnose of mental health or circulatory system diseases), generalized linear models with a log link and Poisson distribution were run to calculate Relative Risks

^b^ All models controlled for pre-baseline covariates (age, gender, ethnicity, sexual orientation, been born in New Zealand, resident in urban area, New Zealand socioeconomic index, occupation, income attribution, educational level, religion, relationship status, political orientation, openness, neuroticism, extraversion, conscientiousness, agreeableness, health locus of control, number of children, hours putting on cosmetics hours commuting, hours looking after children, hours of housework, hours working, pre-baseline screen-based time activities and pre-baseline levels for all outcomes).

^c^ Regression was performed by using the multiple imputed sample of participants under 40 years old (n= 2296).

^d^ For this outcome, only participants married at pre-baseline were analyzed (n=968)

^e^ For this outcome, only participants that weren’t married at pre-baseline were analyzed (n=1328)

**Table S8. Quartiles of total screen-based leisure time (2017) and subsequent outcomes (2019) in the subsample ≥ 40 years old**

|  | **Groups of screen-based leisure time** | | | | | | | | | | | | | | |  |
| --- | --- | --- | --- | --- | --- | --- | --- | --- | --- | --- | --- | --- | --- | --- | --- | --- |
|  | **Quartile 1**  (<18 hours) | **Quartile 2**  (18 to 28 hours) | | | **Quartile 3**  (29 to 42 hours) | | | | | | **Quartile 4**  (>43 hours) | | | | |  |
| **Outcomes by theme** | **β/OR (95%CI)^a,b,c^** | **β/OR (95%CI)^a,b,c^** | ***p*-value** | ***E*-value**  **(lower 95%CI)** | **β/OR (95%CI)^a,b,c^** | | ***p*-value** | | ***E*-value**  **(lower 95%CI)** | | **β/OR (95%CI)^a,b,c^** | | ***p*-value** | ***E*-value**  **(lower 95%CI)** | |  |
| **Psychological wellbeing** |  |  |  |  |  | |  | |  | |  | |  |  | |  |
| Life satisfaction | 0 (ref) | -0.05 (-0.09, -0.01)* | 0.018 | 1.26 (1.10) | -0.01 (-0.05, 0.03) | | 0.643 | | 1.11 (1.00) | | -0.02 (-0.07, 0.02) | | 0.335 | 1.17 (1.00) | |  |
| Self-esteem | 0 (ref) | 0.02 (-0.02, 0.06) | 0.315 | 1.15 (1.00) | 0.01 (-0.03, 0.05) | | 0.728 | | 1.09 (1.00) | | 0.04 (-0.00, 0.09) | | 0.064 | 1.24 (1.00) | |  |
| Body satisfaction | 0 (ref) | -0.01 (-0.06, 0.03) | 0.515 | 1.13 (1.00) | -0.06 (-0.11, -0.01)* | | 0.011 | | 1.30 (1.13) | | -0.09 (-0.14, -0.04)** | | 0.001 | 1.38 (1.22) | |  |
| **Social wellbeing** |  |  |  |  |  |  | |  | |  | |  | | |  | |
| Sense of community | 0 (ref) | -0.00 (-0.05, 0.04) | 0.862 | 1.06 (1.00) | -0.01 (-0.06, 0.04) | | 0.713 | | 1.10 (1.00) | | -0.04 (-0.09, 0.02) | | 0.219 | 1.22 (1.00) | |  |
| Sense of belonging | 0 (ref) | -0.00 (-0.04, 0.04) | 0.955 | 1.03 (1.00) | -0.02 (-0.07, 0.02) | | 0.279 | | 1.17 (1.00) | | 0.02 (-0.03, 0.07) | | 0.359 | 1.17 (1.00) | |  |
| Social support | 0 (ref) | -0.00 (-0.05, 0.04) | 0.844 | 1.07 (1.00) | -0.02 (-0.06, 0.03) | | 0.512 | | 1.13 (1.00) | | -0.00 (-0.06, 0.05) | | 0.852 | 1.07 (1.00) | |  |
| **Social factors** |  |  |  |  |  | |  | |  | |  | |  |  | |  |
| Recently cyberbullied | 1 (ref) | 1.00 (0.62, 1.61) | 0.987 | 1.05 (1.00) | 1.65 (1.04, 2.63)* | | 0.035 | | 1.89 (1.15) | | 1.72 (1.04, 2.84)* | | 0.034 | 1.95 (1.16) | |  |
| Engaged/married/entered a civil union^d^ | 1 (ref) | 0.78 (0.31, 1.96) | 0.601 | 1.51 (1.00) | 0.71 (0.27, 1.91) | | 0.503 | | 1.65 (1.00) | | 1.19 (0.40, 3.55) | | 0.760 | 1.40 (1.00) | |  |
| Divorced/separated^e^ | 1 (ref) | 1.45 (0.80, 2.63) | 0.221 | 1.70 (1.00) | 1.13 (0.57, 2.23) | | 0.725 | | 1.32 (1.00) | | 0.66 (0.29, 1.48) | | 0.310 | 1.77 (1.00) | |  |
| Assaulted/harassed/attacked | 1 (ref) | 1.29 (0.89, 1.87) | 0.174 | 1.53 (1.00) | 1.28 (0.86, 1.90) | | 0.219 | | 1.52 (1.00) | | 1.31 (0.86, 2.02) | | 0.212 | 1.56 (1.00) | |  |
| **Character strengths and prosocial behavior** |  |  |  |  |  | |  | |  | |  | |  |  | |  |
| Volunteering (hours/week) | 0 (ref) | 0.03 (-0.03, 0.08) | 0.369 | 1.18 (1.00) | 0.07 (0.01, 0.13)* | | 0.031 | | 1.32 (1.08) | | 0.10 (0.03, 0.17)* | | 0.004 | 1.42 (1.21) | |  |
| Charity donations ($NZ) | 0 (ref) | -0.02 (-0.08, 0.05) | 0.618 | 1.14 (1.00) | -0.04 (-0.10, 0.03) | | 0.233 | | 1.23 (1.00) | | -0.04 (-0.11, 0.04) | | 0.331 | 1.22 (1.00) | |  |
| Self-control | 0 (ref) | -0.03 (-0.07, 0.01) | 0.200 | 1.18 (1.00) | -0.06 (-0.10, -0.01)* | | 0.010 | | 1.29 (1.12) | | -0.09 (-0.14, -0.04)** | | 0.001 | 1.38 (1.22) | |  |
| **Physical health** |  |  |  |  |  | |  | |  | |  | |  |  | |  |
| Subjective health | 0 (ref) | -0.02 (-0.06, 0.02) | 0.406 | 1.15 (1.00) | -0.04 (-0.09, 0.00) | | 0.060 | | 1.24 (1.00) | | -0.06 (-0.11, -0.01)* | | 0.030 | 1.29 (1.08) | |  |
| Sleep (hours/day) | 0 (ref) | -0.02 (-0.07, 0.03) | 0.358 | 1.16 (1.00) | -0.05 (-0.10, 0.00) | | 0.060 | | 1.26 (1.00) | | -0.07 (-0.12, -0.01)* | | 0.023 | 1.32 (1.10) | |  |
| Body mass index (kg/m^2^) | 0 (ref) | -0.01 (-0.03, 0.02) | 0.656 | 1.08 (1.00) | 0.02 (-0.01, 0.05) | | 0.192 | | 1.15 (1.00) | | 0.06 (0.03, 0.09)** | | <0.001 | 1.29 (1.18) | |  |
| Circulatory system diseases | 1 (ref) | 1.01 (0.90, 1.13) | 0.892 | 1.07 (1.00) | 1.05 (0.94, 1.19) | | 0.380 | | 1.19 (1.00) | | 1.06 (0.94, 1.21) | | 0.336 | 1.21 (1.00) | |  |
| Musculoskeletal system diseases | 1 (ref) | 1.01 (0.77, 1.32) | 0.948 | 1.07 (1.00) | 1.10 (0.83, 1.45) | | 0.509 | | 1.27 (1.00) | | 1.26 (0.93, 1.70) | | 0.135 | 1.49 (1.00) | |  |
| Consequences of external causes | 1 (ref) | 1.38 (0.91, 2.08) | 0.128 | 1.63 (1.00) | 1.57 (1.01, 2.42)* | | 0.044 | | 1.81 (1.08) | | 1.51 (0.93, 2.46) | | 0.099 | 1.76 (1.00) | |  |
| **Mental health** |  |  |  |  |  | |  | |  | |  | |  |  | |  |
| Psychological distress | 0 (ref) | 0.00 (-0.04, 0.04) | 0.984 | 1.02 (1.00) | -0.03 (-0.07, 0.01) | | 0.197 | | 1.19 (1.00) | | -0.01 (-0.06, 0.03) | | 0.566 | 1.13 (1.00) | |  |
| Mental disorders | 0 (ref) | 0.92 (0.78, 1.07) | 0.282 | 1.26 (1.00) | 0.92 (0.78, 1.10) | | 0.365 | | 1.24 (1.00) | | 0.96 (0.80, 1.15) | | 0.657 | 1.17 (1.00) | |  |
| **Health behavior** |  |  |  |  |  | |  | |  | |  | |  |  | |  |
| Alcohol use | 0 (ref) | 0.02 (-0.02, 0.05) | 0.323 | 1.15 (1.00) | 0.02 (-0.02, 0.06) | | 0.320 | | 1.15 (1.00) | | 0.05 (0.01, 0.10)* | | 0.015 | 1.28 (1.11) | |  |
| Current smoking | 1 (ref) | 0.85 (0.62, 1.17) | 0.317 | 1.38 (1.00) | 0.79 (0.57, 1.11) | | 0.181 | | 1.49 (1.00) | | 0.99 (0.70, 1.41) | | 0.969 | 1.06 (1.00) | |  |
| Exercise (hours/week) | 0 (ref) | -0.02 (-0.07, 0.04) | 0.546 | 1.14 (1.00) | 0.04 (-0.02, 0.10) | | 0.148 | | 1.25 (1.00) | | 0.04 (-0.03, 0.11) | | 0.256 | 1.23 (1.00) | |  |

Abbreviations: OR, odds ratio; CI, confidence interval.

* *p* < 0.05 before Bonferroni correction.

** *p* < 0.05 after Bonferroni correction (the *p*-value cutoff for Bonferroni correction is *p* = 0.05/24 outcomes = p < 0.002).

^a^ All continuous outcomes (life satisfaction, self-esteem, sense of community, sense of belonging, social support, volunteering hours, amount of charity donations, self-control, subjective health, psychological distress, hours of sleep, body mass index, body satisfaction, alcohol intake, hours of exercise) were standardized (mean = 0, standard deviation = 1), and the standardized β was calculated through a lineal regression. For binary outcomes with a prevalence <10%, logistical regression models were run to calculate Odds Ratios (been cyberbullied, engaged, married or entered a civil union, divorced or separated , current smoking, diagnose of musculoskeletal system diseases, and consequences of external causes outcomes). For binary outcomes with a prevalence >10% (diagnose of mental health or circulatory system diseases), generalized linear models with a log link and Poisson distribution were run to calculate Relative Risks

^b^ All models were controlled for sociodemographic factors (age, gender, ethnicity, sexual orientation, been born in New Zealand, resident in urban area, New Zealand socioeconomical index, occupation, income attribution, educational level, religion, relationship status, political orientation, openness, neuroticism, extraversion, conscientiousness, agreeableness, health locus of control, number of children, hours putting on cosmetics hours commuting, hours looking after children, hours of housework, hours working, baseline screen-based time activities).

^c^ Regression was performed by using the multiple imputed sample of participants aged 40 years old or more (n= 8789)

^d^ For this outcome, only participants married at pre-baseline were analyzed (n=5681)

^e^ For this outcome, only participants that weren’t married at pre-baseline were analyzed (n=3108)

**References**

VanderWeele, T.J., Ding, P., 2017. Sensitivity analysis in observational research: Introducing the E-Value. Ann Intern Med 167, 268–274. https://doi.org/10.7326/M16-2607
